# Flexible Free‐Standing MoO_3_/Ti_3_C_2_T*_z_* MXene Composite Films with High Gravimetric and Volumetric Capacities

**DOI:** 10.1002/advs.202003656

**Published:** 2020-12-31

**Authors:** Wei Zheng, Joseph Halim, Ahmed El Ghazaly, Ahmed S. Etman, Eric Nestor Tseng, Per O. Å. Persson, Johanna Rosen, Michel W. Barsoum

**Affiliations:** ^1^ Department of Physics, Chemistry and Biology (IFM) Linköping University Linköping 581 83 Sweden; ^2^ Department of Materials Science and Engineering Drexel University Philadelphia PA 19104 USA

**Keywords:** energy density, free‐standing films, hybrid capacitor, MoO_3_ nanobelts, Ti_3_C_2_T*_z_* MXene

## Abstract

Enhancing both the energy storage and power capabilities of electrochemical capacitors remains a challenge. Herein, Ti_3_C_2_T*_z_* MXene is mixed with MoO_3_ nanobelts in various mass ratios and the mixture is used to vacuum filter binder free, open, flexible, and free‐standing films. The conductive Ti_3_C_2_T*_z_* flakes bridge the nanobelts, facilitating electron transfer; the randomly oriented, and interconnected, MoO_3_ nanobelts, in turn, prevent the restacking of the Ti_3_C_2_T*_z_* nanosheets. Benefitting from these advantages, a MoO_3_/Ti_3_C_2_T*_z_* film with a 8:2 mass ratio exhibits high gravimetric/volumetric capacities with good cyclability, namely, 837 C g^−1^ and 1836 C cm^−3^ at 1 A g^−1^ for an ≈ 10 µm thick film; and 767 C g^−1^ and 1664 C cm^−3^ at 1 A g^−1^ for ≈ 50 µm thick film. To further increase the energy density, hybrid capacitors are fabricated with MoO_3_/Ti_3_C_2_T*_z_* films as the negative electrodes and nitrogen‐doped activated carbon as the positive electrodes. This device delivers maximum gravimetric/volumetric energy densities of 31.2 Wh kg^−1^ and 39.2 Wh L^−1^, respectively. The cycling stability of 94.2% retention ratio after 10 000 continuous charge/discharge cycles is also noteworthy. The high energy density achieved in this work can pave the way for practical applications of MXene‐containing materials in energy storage devices.

## Graphical abstract

MoO_3_/Ti_3_C_2_T*_z_* MXene free‐standing films exhibit an excellent combination of high gravimetric and volumetric capacities. The asymmetric supercapacitors with MoO_3_/Ti_3_C_2_T*_z_* anodes and nitrogen‐doped activated carbon cathodes deliver maximum gravimetric and volumetric energy densities of 31.2 Wh kg^−1^ and 39.2 Wh L^−1^, respectively. Binder‐free films with high mass loading bode well for practical applications.

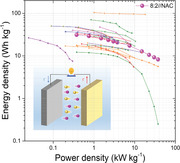

## 1 Introduction

The great demand for portable electronic products and microelectronic devices demands energy and power to be stored in smaller and smaller volumes.^[^ ^1^ ^]^ Electrochemical capacitor are some of the best devices used in electrochemical energy storage because they combine high power densities, long cycle lifes and excellent reliability.^[^ ^2^ ^]^ Electrochemical capacitor are not, however, characterized by high energy densities. To increase the latter—both galvanometric and volumetric—it is imperative to maximize both the volumetric, *C*_v_, and galvanometric, *C*_g_ capacities. However, given that *C*_v_ = *C*_g_*ρ, where *ρ* is the electrode density, for most electrochemical capacitors a tension exists between *ρ* and *C*_g_. Electrode materials like activated carbon, AC, with large specific surface areas and fast ion transfer behavior—have high *C*_g_ values but their low densities lead to low *C*_v_ values. Conversely, dense electrodes are conducive to obtaining high *C*_v_ values, but ion accessibility deteriorates rapidly resulting in lower *C*_g_s’. Therefore, it is nontrivial to design electrodes that have both high *C*_g_ and *C*_v_ values.

Molybdenum oxide (MoO_3_) has attracted much interest as a pseudocapacitive material due to its low cost, nontoxicity, high electrochemical activity and stability.^[^ ^3^ ^]^ Another advantage is its high theoretical capacity (1005 C g^−1^).^[^ ^4^ ^]^ These attributes render it a promising material to achieve both high *C*_g_ and *C*_v_ values. Particularly, the robust layered orthorhombic *α*‐MoO_3_ phase allows fast intercalation and deintercalation of ions in between its layers, without structural collapse. However, the specific capacities of MoO_3_, obtained experimentally are still far below its theoretical capacity. This state of affairs can be traced to its low electronic conductivity, modest reaction kinetics and the limited number of exposed active sites.^[^ ^5^ ^]^

To overcome these problems, various conducting materials such as graphene,^[^ ^6^ ^]^ carbon nanotubes,^[^ ^7^ ^]^ polypyrrole,^[^ ^8^ ^]^ polyaniline,^[^ ^9^ ^]^ and others have been mixed with MoO_3_ to fabricate electrodes. Although this composite approach can effectively enhance *C*_g_ and the rate capabilities of MoO_3_, for the most part the low density of the introduced materials reduces *C*_v_ to some extent. Therefore, new electrodes with high conductivities and higher densities are needed.

The 2D transition metal carbides and/or nitrides, labeled MXenes, with a general formula of M*_n_*_+1_X*_n_*T_z_ (where M in an early transition metal, X is C and/or N, *n* = 1—4, and T*_z_* are surface terminations such as –F, –OH, and –O),^[^ ^10^, ^11^ ^]^ have received tremendous attention for their superior metallic‐like conductivity (≈4000–10 000 S cm^−1^), hydrophilic nature and tunable functional groups, rendering them promising materials in energy storage devices in general, and electrochemical capacitor applications in particular.^[^ ^12^ ^]^ Currently, the most commonly used method to prepare MXenes is to etch their parent MAX phases (short for M*_n_*_+1_AX*_n_*, where *A* is a group A, mostly groups 13 and 14, element) in HF‐containing solutions.^[^ ^10^, ^13^, ^14^ ^]^ The etching stage results in multilayers (MLs). Exfoliating the latter into individual flakes can expose more active sites, facilitating ion storage and thereby boosting the electrochemical performance.^[^ ^13^, ^15^ ^]^

The free‐standing Ti_3_C_2_T*_z_* MXene films assembled from their flakes exhibit *C*_v_ *’*s of the order of 1500 F cm^−3^ in sulfuric acid (H_2_SO_4_).^[^ ^16^ ^]^ These high values can be traced to the relative high electrode densities and excellent intercalation‐based pseudocapacitive energy storage mechanism comparable to the highest values of say, RuO_2_‐based electrodes. Furthermore, if processed correctly, free‐standing MXene films are flexible and can, in principle, be used as flexible electrodes in smart and wearable electronics.^[^ ^17^ ^]^ If the MXene films are simply produced by filtration of colloidal suspensions, it is difficult to keep them from restacking. This restacking limits electrolyte ion penetration to the redox‐active sites and negatively impacts a device's power density. One solution for this problem, is to introduce “spacers” between the MXene interlayers, which serve as “pillars” to prevent restacking.^[^ ^18^, ^19^, ^20^ ^]^ Unfortunately, most reported “spacers” such as reduced graphene oxide (rGO) and carbon nanotubes (CNTs) in MXene‐based composites have low densities and limit the volumetric energy densities.

In this work, we mixed Ti_3_C_2_T*_z_* MXene with MoO_3_ nanobelts, constructing an open, free‐standing film by vacuum filtration. The conductive Ti_3_C_2_T*_z_* nanosheets bridge the MoO_3_ nanobelts and facilitate the rapid transfer of electrons during rapid charge/discharge processes, while the randomly oriented, and interconnected, MoO_3_ nanobelts alleviate the restacking of the Ti_3_C_2_T*_z_* nanosheets. Benefitting from these advantages, we fabricated MoO_3_/Ti_3_C_2_T*_z_* films that exhibited high gravimetric and volumetric capacities with good cyclability. To further improve the energy density, we also fabricated hybrid capacitors, with nitrogen‐doped activated carbon, NAC, as one of the electrodes. The present work provides the guidelines for the production of free‐standing films for energy storage applications.

**Figure 1:**
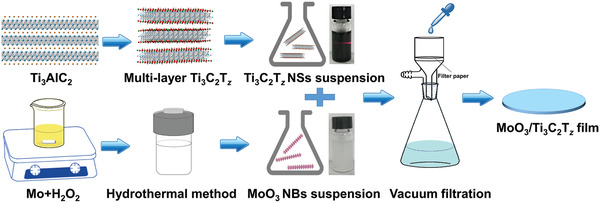
Schematic of the preparation process of the MoO_3_/Ti_3_C_2_T*_z_* film. NB stands for nanobelts and NS stands for nanosheets.

A schematic of the preparation procedure of our MoO_3_/Ti_3_C_2_T*_z_* film electrodes is shown in **Figure** **1**. First, Ti_3_C_2_T*_z_* nanosheets were prepared by etching the Al layers from a Ti_3_AlC_2_ precursor. Second, MoO_3_ nanobelts were prepared using a hydrothermal method. Last, free‐standing MoO_3_/Ti_3_C_2_T*_z_* films were obtained by vacuum filtration of homogeneous suspensions of MoO_3_ and Ti_3_C_2_T*_z_*. We tested five films: pure MoO_3_, pure Ti_3_C_2_T*_z_* and three MoO_3_/Ti_3_C_2_T*_z_* films with mass ratios of 9:1, 8:2, and 7:3. Preliminary work showed that the 8:2 mixture was the best and thus most of the work carried out herein was carried out on that composition.

## 2 Results and Discussion

### 2.1 Phase Composition Analysis and Morphology Characterization

Typical X‐ray diffraction (XRD) patterns and scanning electron microscope (SEM) images of freeze‐dried Ti_3_C_2_T*_z_* nanosheets are shown in Figure S1a,b (Supporting Information), respectively. The Tyndall effect of the Ti_3_C_2_T*_z_* suspension is shown in Figure 1, indicating its good dispersibility in water. The high quality of the Ti_3_C_2_T*_z_* films is reflected in the XRD pattern (Figure S1a, Supporting Information) where peaks that do not belong to Ti_3_C_2_T*_z_* are absent. The conductivities of Ti_3_C_2_T*_z_* films at room temperature (RT) were calculated from the sheet resistances, obtained by a four‐point probe DC measurement, to be 4190 ± 30 S cm^−1^ (Figure S2, Supporting Information). This value is typical of good quality filtered MXene films.^[^ ^21^ ^]^

Similarly, XRD patterns of the MoO_3_ nanobelts (Figure S3a, Supporting Information) confirm their high purity. The lengths of nanobelts can reach tens of micrometers and thicknesses of up to 35 nm, as shown in Figure S3b,c (Supporting Information), resulting in a high length‐to‐width ratio typical of such nanobelts.^[^ ^22^ ^]^ The favorable growth of MoO_3_ along the [001] direction forming this belt‐like structure is determined by its highly anisotropic crystal structure, and has been observed in previous reports.^[^ ^5^, ^23^ ^]^

**Figure 2:**
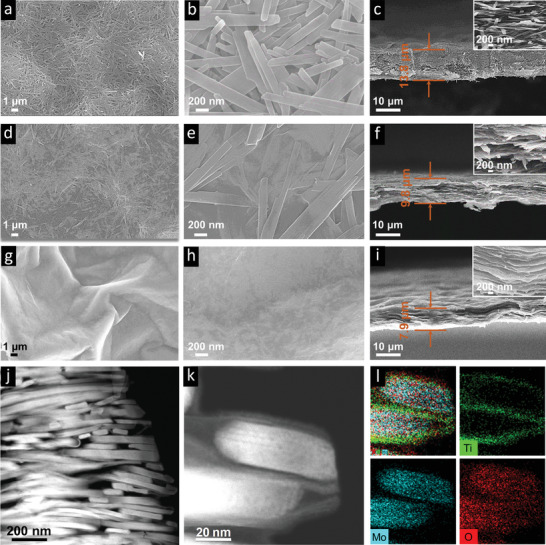
SEM images of MoO_3_ in plan‐view a,b) and cross‐section c) of the 8:2 MoO_3_/Ti_3_C_2_T*_z_* composite in plan‐view d,e) and cross‐section f), and finally of the Ti_3_C_2_T*_z_* film in plan‐view (g,h) and cross‐section i). Low‐magnification cross‐sectional STEM images of the 8:2 film j) and at medium magnification k) with EDX elemental maps of Ti (green), Mo (blue), and O (red) shown in l).

Digital photos of the various films fabricated here are shown in Figure S4 (Supporting Information). Despite not using any binders, all films are quite flexible (inset in Figure S4 in Supporting Information). SEM plan‐view images of three fabricated films are displayed in **Figure** **2**. The MoO_3_ film (Figure 2a,b), shows a mesh‐like structure, with intertwined nanobelts. The Ti_3_C_2_T*_z_* film (Figure 2g,h) exhibits a rugged surface with wrinkles caused by nanosheet restacking. The 8:2 composite films (Figure 2d,e), on the other hand, show a relative flat, loose structure in which the Ti_3_C_2_T*_z_* nanosheets interconnect the MoO_3_ belts, yielding a more robust film. Meanwhile, the randomly oriented and interconnected MoO_3_ nanobelts alleviate the restacking of the Ti_3_C_2_T*_z_* nanosheets, opening more active regions for ion adsorption/desorption. Not surprisingly, the addition of Ti_3_C_2_T*_z_* to MoO_3_ enhanced the overall conductivity of the films by six orders of magnitude (Figure S2, Supporting Information).

The corresponding SEM cross‐sectional images of the same three films are shown in Figure 2c,f,i. Given that the mass loadings of all films were nearly the same (≈2.2 mg cm^−2^) we conclude from the SEM micrographs that the Ti_3_C_2_T*_z_* film (Figure 2i) is the densest, and the MoO_3_ film (Figure 2c) is the least dense, with the 8:2 composite film in between (Figure 2f). The thickness of the latter is ≈10 µm (Figure 2f). The morphologies of the other ratio films are shown in Figure S5 (Supporting Information). Consistent with the results shown in Figure 2, the film thicknesses decrease with increasing Ti_3_C_2_T*_z_* content.

Scanning transmission electron microscope (STEM) cross‐sectional images and corresponding energy dispersive spectroscope (EDS) elemental mapping of the 8:2 film are shown in Figure 2j–l. The low magnification STEM image of an 8:2 film (Figure 2j) displays a more or less ordered structure out of plane. In plane, however, (Figure 2d,e), the order is less good, probably due to the rapid filtration process of the well mixed suspensions. Another reason may be ascribed to a possible reaction between MoO_3_ and Ti_3_C_2_T*_z_* during processing (see XPS results below). As seen from cross‐sectional STEM, the MoO_3_ nanobelts (Figure 2k) exhibit an average thickness of ∼30–40 nm, which agrees well with the SEM images (Figure S3, Supporting Information). The titanium (green), molybdenum (blue), and oxygen (red) EDX elemental maps clearly show that the MoO_3_ nanobelts are effectively percolated by Ti_3_C_2_T*_z_* nanosheets with a homogeneous stacking structure, enabling high conductivity and excellent flexibility (Figure 2k). A lattice resolved STEM image from the same area is shown in Figure S6 (Supporting Information). The image clearly resolves the interface between the Ti_3_C_2_T*_z_* nanosheets and MoO_3_, as well as the organized stacking of the MXene sheets.

**Figure 3:**
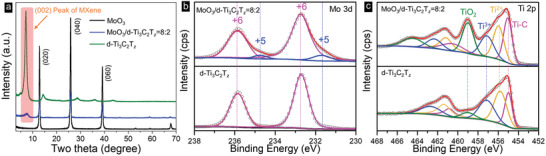
a) XRD patterns of MoO_3_, 8:2, and Ti_3_C_2_T*_z_* films. XPS spectra of b) Mo 3d of MoO_3_ in pure MXene (bottom) and 8:2 films (top), and c) Ti 2p of Ti_3_C_2_T*_z_* (bottom) and 8:2 (top) films.

Typical XRD patterns of the two‐end member and a 8:2 composite films are shown in **Figure** **3**a. The strong intensity of (020), (040) and (060) peaks in the MoO_3_ pattern (bottom) indicates the strong preferred orientation of the ribbons after vacuum filtration. The absence of a peak at ≈ 60° at 2*θ* and the intense (002) peak for the pure Ti_3_C_2_T*_z_* film (top) also indicates that the flakes are oriented. From the (002) peak position at 2*θ* = 7.2°, we calculated an interlayer spacing of 1.2 nm. The XRD pattern of the 8:2 composite film (middle) confirms that, at least at this scale, there were no side reactions between the two end members. The XRD patterns of the other ratios are shown in Figure S7 (Supporting Information). Intriguingly, all the (0*l*0) peaks of MoO_3_ in MoO_3_/Ti_3_C_2_T*_z_* films have a slight shift to smaller 2*θ* angles compared with the pure MoO_3_ film. The shift increases with increasing Ti_3_C_2_T*_z_* content. At this time, the origin of this shift is unknown.

X‐ray Photoelectron Spectroscopy (XPS) spectra of the 8:2 composite film in the Mo 3d and Ti 2p regions are shown in Figure 3b,c, respectively. The fitting results are listed in Tables S1 and S2 (Supporting Information). The peaks at 232.7 and 235.9 eV in Figure 3b represent Mo^6+^ 3d_5/2_ and 3d_3/2_, respectively.^[^ ^24^ ^]^ Small, new Mo peaks at 231.7 and 234.8 eV appear in the composite film's XPS spectra (Figure 3b, top). These peaks are assigned to Mo^5+^ 3d_5/2_ and 3d_3/2_, respectively.^[^ ^6^ ^]^ When the pure and composite films Ti 2p regions are compared (Figure 3c), it is obvious that the fraction of Ti^4+^ increased in the latter. Based on these results, it is reasonable to conclude that when the two suspensions are mixed, a fraction of the Mo^6+^ ions are reduced by Ti ions in the Ti_3_C_2_T*_z_*. This somewhat unexpected result is nonetheless advantageous because it is established that reduced MoO_3_ with oxygen vacancies exhibits better electrochemical performance compared with pure MoO_3_.^[^ ^4^, ^5^, ^24^ ^]^

### 2.2 Electrochemical Characterization

**Figure 4:**
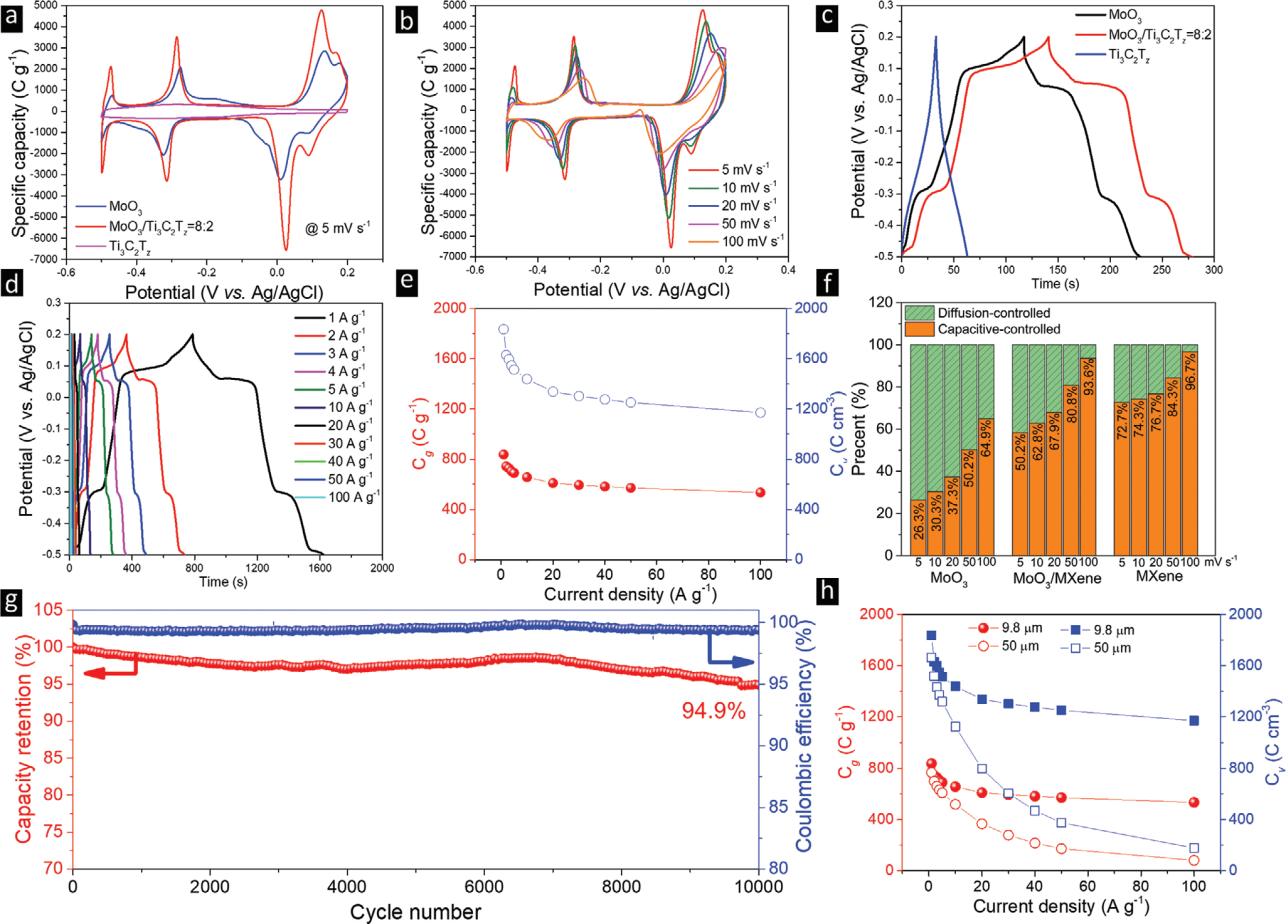
a) Comparison of CV curves at 5 mV s^−1^ for MoO_3_, 8:2, and Ti_3_C_2_T*_z_* films; b) CV curves of 8:2 films at scan rates from 5 to 100 mV s^−1^; c) comparison of GCD curves at 5 A g^−1^ for three films in (a); d) GCD curves of composite film at current densities between 1 and 100 A g^−1^; e) specific gravimetric (red, left *y*‐axis) and volumetric (right blue axis) capacities of composite film as a function current densities; f) capacitive‐ and diffusion‐controlled contributions for the three films in (a), at different scan rates; g) cycling stability of 8:2 film measured at 50 A g^−1^ for 10 000 cycles; h) specific (left y‐axis) and volumetric (right y‐axis) capacity comparisons of ≈10 and ≈51 µm films at different current densities.

The *C*_g_ and *C*_v_ values of five electrodes: the two end members and three composite ones are compared in Figure S8a,b (Supporting Information), respectively. From these results it is clear that the 8:2 electrode is the best. This is consistent with the morphological characteristics shown in Figure 2 and Figure S5 (Supporting Information). **Figure** **4**a compares the CV curves for the end members and the 8:2 electrode, at a scan rate of 5 mV s^−1^. The pure Ti_3_C_2_T*_z_* film shows an asymmetric oval‐like cycle with no peaks, consistent with a pseudocapacitive performance in H_2_SO_4_ electrolyte by H^+^ intercalation/deintercalation (see Figure 4a and Figure S9 in the Supporting Information).^[^ ^25^ ^]^ The MoO_3_ and composite films (Figure 4a,b; Figures S10–S12, Supporting Information), have four pairs of redox peaks contributing to the overall specific capacity. These redox peaks can be attributed to the insertion/extraction of H^+^ in combination with the reduction and oxidation of Mo (Mo^6+^ ↔ Mo^5+^ ↔ Mo^4+^).^[^ ^26^, ^27^ ^]^ The whole process can be expressed by the following somewhat generalized equations

(1) $$xH^{+}+xe^{-}+\mathrm{Mo}O_{3}↔H_{x}\mathrm{Mo}O_{3}$$

(2) $$yH^{+}+ye^{-}+H_{x}\mathrm{Mo}O_{3}↔H_{x+y}\mathrm{Mo}O_{3}$$

Crucially, the redox peaks are clearly observed even at scan rates of 100 mV s^−1^ in Figure 4b, reflecting the fast H^+^ insertion/extraction and concomitant Faradaic reactions. With increasing scan rates, the oxidation peaks shift to higher potentials and the reduction peaks shift to lower potentials, which is commonly observed in Faradic reactions and is mainly explained by the electrode/concentration polarization at high scan rates, leading to a larger IR drop and sluggish redox reactions as ionic diffusion and electron transfer are slowed down, further increasing the differences between the oxidation/reduction peaks.

The GCD curves of the three films are compared in Figure 4c. At 5 A g^−1^, the discharge time of the composite film is the longest, meaning it stored—in accordance with the CV results—the largest charge as compared to the pure MoO_3_ and Ti_3_C_2_ films. All the GCD curves show symmetric charge/discharge processes, indicating good reaction reversibility. No obvious plateaus can be observed in the Ti_3_C_2_T*_z_* film, while both the MoO_3_ and the composite films exhibit four pairs of plateaus, corresponding to the redox peaks in their CV curves. Regardless of the current density, all the charge/discharge curves for the composite electrode are symmetric (Figure 4d). The CV curves and GCD curves of the other composite films are plotted in Figures S11 and S12 (Supporting Information) and are quite similar to those of the 8:2 electrodes, indicating the same electrochemical processes are occurring in all. The gravimetric and volumetric capacities of the 8:2 film are displayed in Figure 4e, as a function of current densities from 1 to 100 A g^−1^. More specifically, capacities of 837 C g^−1^ (233 mA h g^−1^) or 1836 C cm^−3^ (510 mA h cm^−3^) were obtained at 1 A g^−1^. When the current density increased to 100 A g^−1^, 63.8% of the rate capability was retained. The high current density GCD curves for the 5 films are compared in Figure S13 (Supporting Information).

Figure 4f and Figure S14 (Supporting Information), compare the capacitive‐ and diffusion‐controlled contributions to the overall capacity for the end members and the 8:2 electrode. In all cases, not surprisingly, the capacitive component increases with increasing scan rates. For the MoO_3_ film, the high values for the diffusion‐controlled contribution imply that bulk reactions are slower than surface electrochemical reactions. For the pure Ti_3_C_2_T*_z_* film, the electrochemical reaction is controlled by surface reactions, implying relatively high charge transfer rates. For the composite film, the capacitive‐controlled contribution is improved compared to the MoO_3_ film at the same scan rate, indicating its good rate capability because of its excellent electrical conductivity and shortened diffusion/transport paths. The Nyquist plots obtained on the various films are plotted in Figure S15 (Supporting Information). The internal resistances (*R*_s_) and charge transfer resistances (*R*_ct_) of the 8:2 composite films are reduced significantly compared with pure MoO_3_. After 10 000 cycles at 50 A g^−1^ (Figure 4g), ≈94.9% of the initial capacity was retained.

Maximizing an electrodes’ mass loading, is crucial in maximizing a device's specific energy storage. For example, a device's performance will drop 50%, when a mass loading of 10 mg cm^−2^ is compared to say a loading of 1–3 mg cm^−2^.^[^ ^28^ ^]^ To get a sense of what penalty increasing the electrodes’ thicknesses and mass loadings would have on our capacities, we compared the responses of a ≈50 µm thick electrode with a mass loading of 11 mg cm^−2^ (Figure S16, Supporting Information) with a ≈10 µm thick one with a mass loading of 2.2 mg cm^−2^. Figure 4h shows the results: the thick film still delivers 767 C g^−1^ (213 mA h g^−1^) and 1664 C cm^−3^ (462 mA h cm^−3^) at 1 A g^−1^. At 50 A g^−1^, the values are 173 C g^−1^ and 375 C cm^−3^. It is worth noting that the thick electrodes were also quite flexible (Figure S17, Supporting Information). The CV and GCD curves of the thick film are shown in Figure S18a,b (Supporting Information). Their shapes are in good agreement with the thin film (≈10 µm), indicating the nature of the reaction does not change by increasing the mass loading. Furthermore, the negligible increments in *R*_s_ and *R*_ct_ in Figure S18c (Supporting Information), compared with ≈10 µm thin film, makes it possible to consider such films for practical applications.

**Figure 5:**
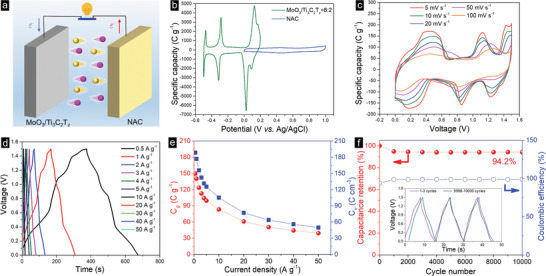
Electrochemical characteristics of 8:2//NAC hybrid capacitor. a) Schematic diagram of hybrid capacitor; b) CV curves at 5 mV s^−1^; c) CV curves at scan rates from 5 to 100 mV s^−1^; d) GCD curves at different current densities from 1 to 50 A g^−1^; e) Specific capacities at different current densities; and f) Cycling stability of 8:2//NAC capacitor measured at 10 A g^−1^ for 10 000 cycles.

One problem with aqueous electrochemical capacitor is their relatively narrow voltage window that, in turn, results in lower energy densities. To roughly double this window, we assembled a hybrid capacitor, with the 8:2 composite film as the negative electrode and a NAC film as the positive electrode, with 3m H_2_SO_4_ as the electrolyte (**Figure** **5**a). The morphologies of the NAC particles and films are shown in Figures S19 and S20 (Supporting Information). The electrochemical performance of this electrode is shown in Figure S21 (Supporting Information).

Figure 5b displays the CV curves, at 5 mV s^−1^, of the 8:2 composite and NAC electrodes separately. The former exhibits four pairs of redox peaks and has a potential window from −0.5 to 0.2 V. The NAC's potential window is from 0 to 1 V. It follows that the window of our hybrid capacitor is from 0 to 1.5 V. Figure S22 (Supporting Information) plots the CV curves from 0 to 1.6 V, from which it is clear that at 1.6 V a noticeable sharp current increase is observed most probably due to electrolyte decomposition/water splitting.^[^ ^29^ ^]^ Therefore, we limited our potential window to 0–1.5 V.

When the CV curves of the hybrid capacitor are plotted (Figure 5c), three pairs of redox peaks are observed. Increasing the scan rates does not appear to distort the curves, indirectly demonstrating the fast charge/discharge processes and excellent rate performances. The GCD curves, at various current densities, are plotted in Figure 5d, where the plateaus observed correspond to the CV curves. The capacities calculated, based on the discharge times, are shown in Figure 5e. The as‐prepared device shows good rate performance: even at 50 A g^−1^, it still delivers 39.5 C g^−1^ and 49.7 C cm^−3^ (Figure 5e; Figure S23, Supporting Information). In comparison, the MoO_3_//NAC and Ti_3_C_2_T*_z_*//NAC hybrid capacitor only store 27.1 C g^−1^ (32.9 C cm^−3^) and 1.2 C g^−1^ (1.6 C cm^−3^) at the same current density (see Figures S24 and S25 in the Supporting Information).

When the cycling stability of the 8:2//NAC hybrid capacitor are plotted (Figure 5f), 94.2% of the capacity is retained after 10 000 charge/discharge cycles at 10 A g^−1^, exhibiting outstanding cycling performance. The XRD patterns and SEM images of the 8:2 films after cycling in the hybrid capacitor show no obvious changes (Figure S26, Supporting Information), confirming its good cycling stability and robust structure, which bodes well for practical applications.

**Figure 6:**
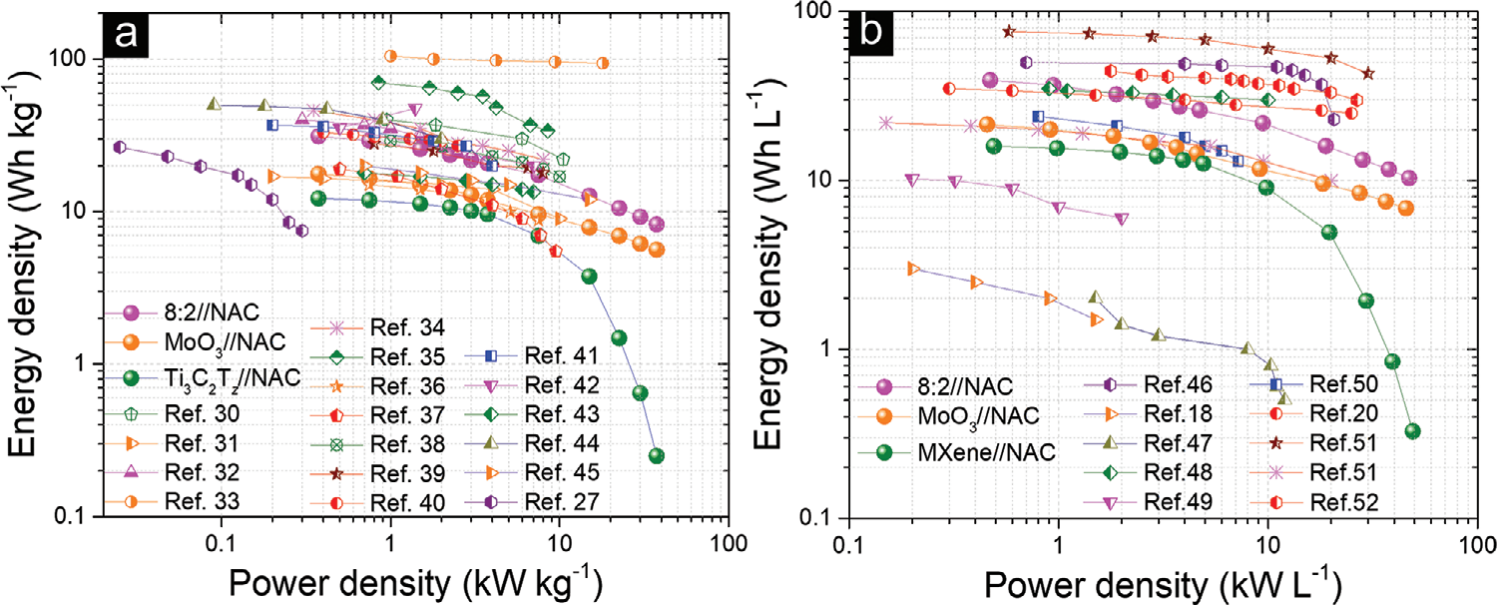
Ragone plots for 8:2//NAC hybrid capacitors and previously reported values: a) specific values compared with mass loadings of ≈2 mg cm^−2^, such as MnCo_2_O_4_@NiMoO_4_//AC in KOH,^[^ ^30^ ^]^ Ti_3_C_2_T*_z_*//PEDOT@rGO in H_2_SO_4_,^[^ ^31^ ^]^ NiO//rGO in KOH^[^ ^32^ ^]^ Co(OH)_2_//N‐doped graphene in KOH,^[^ ^33^ ^]^ Ti_3_C_2_T*_z_*/Ni‐Co‐Al‐LDH//AC in KOH,^[^ ^34^ ^]^ NiCo_2_S_4_/Ti_3_C_2_T*_z_*//AC in KOH,^[^ ^35^ ^]^ TC‐9//Ti_3_C_2_T*_z_* in KOH,^[^ ^36^ ^]^ Ni–S/Ti_3_C_2_T*_z_*//Ti_3_C_2_T*_z_* in KOH,^[^ ^37^ ^]^ ML‐80//MG‐5 in KOH,^[^ ^38^ ^]^ Ti_3_C_2_T*_z_*/CoS_2_ (CCH)//rGO in KOH,^[^ ^39^ ^]^ CoNi_2_S_4_//AC in KOH,^[^ ^40^ ^]^ Ni*_x_*Co_3−_ *_x_*O_4_//AC in KOH,^[^ ^41^ ^]^ Co_3_O_4_//AC in KOH,^[^ ^42^ ^]^ CuO//AC in KOH,^[^ ^43^ ^]^ Ni(OH)_2_/CNT/NF//AC in KOH,^[^ ^44^ ^]^ Ni_3_S_2_/MWCNT‐NC//AC in KOH,^[^ ^45^ ^]^ MoO_3_//PBA hydrogen‐ion battery in H_3_PO_4_;^[^ ^27^ ^]^ b) volumetric values compared with mass loadings of ≈1 mg cm^−2^, such as Ti_3_C_2_T*_z_*//PANI/Ti_3_C_2_T*_z_* in H_2_SO_4_,^[^ ^46^ ^]^ EGMX1:3//EGMX1:3 in H_2_SO_4_,^[^ ^18^ ^]^ V_2_CT*_z_*//V_2_CT*_z_* in LiCl,^[^ ^47^ ^]^ AQS/G‐1//RuO_2_/G in H_2_SO_4_,^[^ ^48^ ^]^ Ti_3_C_2_T*_z_*/MWCNT//rGO_T_ in H_2_SO_4_,^[^ ^49^ ^]^ MXene/P‐100‐H//rGO in H_2_SO_4_,^[^ ^50^ ^]^ Mo_1.33_C/PEDOT:PSS//Mo_1.33_C/PEDOT:PSS in H_2_SO_4_,^[^ ^20^ ^]^ N‐doped Ti_3_C_2_T*_z_*//N‐doped Ti_3_C_2_T*_z_* in H_2_SO_4_,^[^ ^51^ ^]^ and MoO_3_/Ti_3_C_2_T*_z_*//MoO_3_/Ti_3_C_2_T*_z_* in H_2_SO_4_.^[^ ^52^ ^]^

Based on the mass of the active materials in the negative and positive electrodes, the maximum gravimetric energy and power densities (as shown in **Figure** **6**a,b) calculated from the GCD curves are 31.2 Wh kg^−1^ (at 0.5 A g^−1^) and 37.5 kW kg^−1^ (at 50 A g^−1^), respectively. Correspondingly, the maximum volumetric energy and power densities are 39.2 Wh L^−1^ and 47.1 kW L^−1^, respectively.

At this juncture it is useful to place our results in perspective. Compared with previous reports on a large number of different systems and mass loadings (Figures S27–29 and Table S3, Supporting Information), our *C*_g_ and *C*_v_ values hold their own and are higher than a majority of reported values for MXene‐containing electrochemical capacitors.

As far as we are aware, these is only one other, quite recent, paper on MoO_3_ nanobelts Ti_3_C_2_T*_z_* composite electrochemical capacitor electrodes.^[^ ^52^ ^]^ Wang et al. fabricated 20 wt% MoO_3_ nanobelts—balance Ti_3_C_2_T*_z_* (≈ 1 mg cm^−2^ mass loading)—and obtained a volumetric capacity of 1635 C cm^−3^ at 1 A g^−1^. They also reported an energy density of 13.4 Wh kg^−1^ or 44.6 Wh L^−1^. We obtained higher numbers (1836 C cm^−3^ at 1 A g^−1^). Importantly, our mass loading (2.2 mg cm^−2^) is twice as large. Similarly, our hybrid capacitor produced an energy density of 31.2 Wh kg^−1^ or 39.2 Wh L^−1^.

A perusal of the Ragone plots (Figure 6a,b) is also instructive in that they demonstrate that the energy and power densities of our hybrid capacitor are competitive, especially the volumetric values. It is worth noting that our combination of energy densities, at high powers, is excellent.

The self‐discharge rate of our capacitor is compared to previous work in Figure S30 (Supporting Information). Clearly, our discharge rate would have to be greatly enhanced before any applications can be contemplated.

## 3 Summary and Conclusions

In summary, we vacuum filtered, flexible, highly conducting, free‐standing MoO_3_/Ti_3_C_2_T*_z_* films. These films were then tested as electrochemical capacitor electrodes. Films with an 8:2 MoO_3_/Ti_3_C_2_T*_z_* mass ratio, respectively, stored 837 C g^−1^ (1836 C cm^−3^) at 1 A g^−1^ at a mass loading of >2 mg cm^−2^. When the mass loading was increased to a commercial level (>10 mg cm^−2^), these composite films still stored 767 C g^−1^ (1664 C cm^−3^) at 1 A g^−1^.

A hybrid capacitor based on MoO_3_/Ti_3_C_2_T*_z_* and nitrogen‐doped activated carbon delivered an energy density of 31.2 Wh kg^−1^, corresponding to a volumetric energy density of 39.2 Wh L^−1^.

The excellent electrochemical performance can be ascribed to the following reasons: i) the high conductivity of Ti_3_C_2_T*_z_* provides a path for electrons, allowing for rapid charge/discharge processes; ii) the MoO_3_ nanobelts alleviate the restacking of Ti_3_C_2_T*_z_*, exposing more area for energy storage; iii) the reduction of the MoO_3_ due to the introduction of Ti_3_C_2_T*_z_* could further increase the free carrier concentration and generate more exposed active sites, resulting in fast charge storage kinetics and higher capacities; iv) the open and robust structures of our 8:2 electrodes allow the electrolyte to penetrate and wet the electrodes, enhancing the specific capacity and prolonging the cycling performance; and v) the synergetic effects of the high density and high capacity of Ti_3_C_2_T*_z_* and MoO_3_ enable high gravimetric and volumetric performance.

## 4 Experimental Section

All materials and chemical agents were purchased from Sigma‐Aldrich and used as purchased unless otherwise noted.

##### Materials Preparation—MoO_3_ Nanobelts

0.5 g of Mo powder (1–5 µm, > 99.9%) was added into 10 mL of H_2_O_2_ (30%) while vigorously stirring for 0.5 h at room temperature (RT). Then 20 mL of deionized water was added into the above solution, followed by stirring for another 0.5 h. The obtained luminous yellow solution was transferred into a 50 mL Teflon‐lined stainless‐steel autoclave and heated to 140 °C for 24 h. After the reaction was complete, the resulting white precipitate was washed alternately with 30 mL of ethyl alcohol and deionized water three times, respectively, by centrifuging 2 min at 6000 rpm. Finally, the as‐prepared powder was dried at 70 °C for 24 h.

##### Materials Preparation—MXene Suspensions

Delaminated Ti_3_C_2_T*_z_* suspensions was prepared by etching of Ti_3_AlC_2_ powders (Lab‐made, particle size <60 µm; the synthesis of the Ti_3_AlC_2_ precursor powders can be found in ref. ^[^ ^12c^ ^]^) followed by an ultrasonic exfoliation process. Specifically, 0.5 g of Ti_3_AlC_2_ powders was slowly added to a 10 mL mixture solution containing 12 m HCl (Fisher, technical grade) and 2.3 m of LiF (Alfa Aesar, >98%) in a Teflon bottle. Prior to adding the Ti_3_AlC_2_ powder, the latter was placed in an ice bath. The mixture was kept in the ice bath for 0.5 h, to avoid initial overheating that can result from the exothermic nature of the reaction. The bottle was then placed on a hot plate and magnetically stirred in an oil bath and held at 35 °C for 24 h, after which the mixture was washed through three cycles with 40 mL of 1 m HCl, followed by three cycles with 40 mL of 1 m LiCl (Alfa Aesar, >98%). The mixture was then washed through cycles with 40 mL of deionized water until the supernatant pH reached was ≈6. After washing, 45 mL of deionized water was added and deaerated by bubbling N_2_ gas through it after which it was sonicated using an ultrasonic bath for 1 h. The resulting suspension was centrifuged 20 min at 2000 rpm. The supernatant concentration was measured by weighting the film filtrated from a known volume of suspension.

##### Materials Preparation—MoO_3_/Ti_3_C_2_T_z_ Free‐Standing Films

To fabricate composite films, first 1 mg mL^−1^ of d‐Ti_3_C_2_ and 1 mg mL^−1^ of MoO_3_ suspensions were prepared. Then, 4 mL of former was slowly added into 16 mL of the latter. After 5 min of vigorous magnetic stirring, the homogeneous mixture suspension was vacuum filtered through a membrane (Celgard 3501, 0.064 µm pore size, Celgard LLC) to produce MoO_3_/Ti_3_C_2_T*_z_* films.

As noted above three films were fabricated with MXene mass fraction percentages of 10, 20, and 30 wt%, balance MoO_3_. After some preliminary experiments it was established the 8:2 MoO_3_/Ti_3_C_2_T*_z_* films yielded the best results and are thus the ones that we studied most carefully. These films were also easily peeled off from the porous membrane after drying in air. For comparison's sake, pure MoO_3_, Ti_3_C_2_T*_z_*, 9:1, and 7:3 films were also prepared using the same procedure outlined above. The compositions of the various films are listed in Table S4 (Supporting Information). The obtained films were stored in a glove box (Jacomex GP Campus T2, 99.9999% purity argon, Ar, atmosphere, H_2_O <1 ppm, O_2_ <1 ppm) to prevent their oxidation.

##### Materials Preparation—Nitrogen Activated Carbon (NAC) Electrode

To prepare the NAC electrodes, 10 g of urea—used as a nitrogen, N, source—and 0.4 g of activated carbon (AC) were added to 30 mL of deionized water and stirred for 0.5 h at RT. The obtained suspension was transferred into a 50 mL Teflon‐lined stainless‐steel autoclave and heated in an oven to 180 °C for 24 h. After the reaction was completed, the product was washed with 30 mL of deionized water at least ten times by centrifuging for 2 min at 6000 rpm. Finally, the as prepared sample was dried at 70 °C for 24 h.

##### Materials Characterization

XRD was used to characterize the phases present using a diffractometer (PANalytical X'Pert) with Cu K*α* (40 kV, 40 mA). The 2*θ* range was from 3° to 70° in a continuous mode. The step size and dwell times were 0.008° and 40 s, respectively.

A SEM (LEO 1550 Gemini) and a STEM (The Linköping double corrected FEI Titan^3^ 60‐300), equipped with a Super‐X quadruple EDS detector were used to examine the microstructure, morphologies and elemental compositions of the samples.

XPS measurements were performed on free standing films by a surface analysis system (Kratos AXIS Ultra^DLD^, Manchester, UK) using monochromatic Al K*α* (1486.6 eV) radiation. The X‐ray beam irradiated the surface of the sample at an angle of 45°, with respect to the surface and provided an X‐ray spot of 300 × 800 µm. Charge neutralization was performed using a coaxial, low energy (≈0.1 eV) electron flood source to avoid shifts in the recorded binding energies (BE). XPS spectra were recorded for Ti 2p and Mo 3d. The analyzer pass energy used for all the regions was 20 eV with a step size of 0.1 eV. The BE scale of all XPS spectra was referenced to the adventitious carbon (284.8 eV).

The sheet resistances, *R*_s_, of select films were measured by a four‐point probe instrument (Jandel RM3000, UK). Each film was measured in at least five different locations. The *R*_s_ was converted to a conductivity, by multiplying by the film thickness. The latter was measured from the cross‐sectional images of the films obtained by SEM.

##### Electrochemical Measurements

For the three‐electrode configuration, an overcapacitive activated carbon (AC) disc (YP50F, Kuraray, Japan) was used as a counterelectrode, Ag/AgCl in saturated KCl as a reference electrode, and small, 4 mm diameter MoO_3_/d‐Ti_3_C_2_ discs were used as working electrodes. The electrolyte was 3 m H_2_SO_4_. All cells used glassy carbon (CHI Instruments, China) as current collectors and polypropylene membranes (Celgard 3501) as separators.

Three electrode half‐cells were assembled in plastic Swageloks and tested using a potentiostat (VSP, Biologic, France). Before measurements, the cell was precycled at 20 mV s^−1^ for 100 cycles to stabilize the performance. The thickness, mass loading, and packing density of the 8:2 films were ≈10 µm, ≈2.2 mg cm^−2^, and ≈2.2 g cm^−3^, respectively. The respective values for the MoO_3_ films were ≈13.8 µm, ≈2.2 mg cm^−2^, and ≈1.6 g cm^−3^, respectively. For the d‐Ti_3_C_2_ films, they were ≈7.9 µm, ≈2.3 mg cm^−2^, and ≈2.9 g cm^−3^, respectively.

The two‐electrode hybrid capacitor cell was comprised of a MoO_3_/d‐Ti_3_C_2_ negative electrode and an NAC positive electrode. The average packing density based on the whole active materials (negative and positive) is 1.26 g cm^−3^. The AC and NAC discs were prepared as follows: 90 wt% of the active materials were mixed uniformly with the aid of deionized water with 10 wt% poly(tetrafluoroethylene) (PTFE) binder using a mortar and pestle. The slurry was then rolled into flat films using a glass cylinder with a diameter of ≈7 cm. At last, 5 mm (for AC) and 4 mm (for NAC) diameter discs were punched from the films and dried at 70 °C for 24 h.

The procedure to calculate the various capacities are outlined in the Supporting Information, together with the procedure to estimate the capacitive‐ and diffusion‐controlled contributions to total charge.

## Conflict of Interest

The authors declare no conflict of interest.

## Supporting information

Supporting Information
