# Supplementary material for: Flexible Free‐Standing MoO3/Ti3C2Tz MXene Composite Films with High Gravimetric and Volumetric Capacities
Source: Adv Sci (Weinh). 2020 Dec 31;8(3):2003656. doi: 10.1002/advs.202003656 (PMC7856882; doi:10.1002/advs.202003656)
Supplement: Supplementary file 1 — Supporting Information [file ADVS-8-2003656-s001.pdf]

WILEY-VCH

© 2020 Wiley-VCH GmbH

ADVANCED SCIENCE
Open Access

Supporting Information

for *Adv. Sci.,* DOI: 10.1002/advs.202003656

Flexible Free-standing $MoO_3/Ti_3C_2T_z$ MXene Composite Films with High Gravimetric and Volumetric capacities

Wei Zheng, Joseph Halim, Ahmed El Ghazaly, Ahmed S. Etman, Eric Nestor Tseng, Per O. Å. Persson, Johanna Rosen and Michel W. Barsoum**

Supporting Information

Flexible Free-standing $MoO_3/Ti_3C_2T_z$ MXene Composite Films with High Gravimetric and Volumetric capacities

Wei Zheng[1], Joseph Halim[1], Ahmed El Ghazaly[1], Ahmed S. Etman[1], Eric Nestor Tseng[1], Per O. Å. Persson, Johanna Rosen*[1] and Michel W. Barsoum*[2]

1. Thin Film Physics, Department of Physics, Chemistry and Biology (IFM), Linköping University, SE-581 83 Linköping, Sweden.
2. Department of Materials Science and Engineering, Drexel University, Philadelphia, Pennsylvania 19104, USA.

*Email: johanna.rosen@liu.se; barsoumw@drexel.edu

Procedures to calculate C_g and C_v:

The electrode's gravimetric specific capacity, C_g, was calculated from the galvanostatic charge/discharge (GCD) curves according to the following equation:

$$C_g = I\Delta t/m \quad \text{(S1)}$$

where I is the discharge current, Δt is the discharging time, and m is the mass of the active materials in the electrodes. Note here no binders or conducting additives were used in any of the MXene or MoO_3 containing films.

The volumetric capacity (C_v) was obtained from the following equation:

$$C_v = \rho C_g \quad \text{(S2)}$$

where ρ is the density of the films, which was calculated assuming:

$$\rho = m/Sd \quad \text{(S3)}$$

where m, S and d are the mass, area and thickness of the electrode, respectively.

For the hybrid capacitor devices, the mass ratio of the negative to positive electrodes was decided based on charge balance theory (q_+=q_-). The charge stored (q) by each electrode was estimated assuming:

$$q = C_g \times m \quad \text{(S4)}$$

The gravimetric energy, E_g, and power, P_g, densities were calculated assuming:

$$E_g = C_g U/2 \quad \text{(S5)}$$

$$P_g = E_g/\Delta t \quad \text{(S6)}$$

Where U is the output voltage of the ASC devices.

The volumetric energy, E_v, and power densities, P_v, were calculated assuming:

$$E_v = \rho E_g \quad \text{(S7)}$$

$$P_v = E_v/\Delta t \quad \text{(S8)}$$

Estimation of capacitive- and diffusion-controlled contributions to total charge

The capacitive-controlled and the diffusion-controlled contributions to the total charge storage were calculated based on the Ref 1.[1]

In brief, the current response i at a certain potential (V) is given by k_1v+$k_2v^{1/2}$, where k_1v

and $k_2v^{1/2}$ correspond to capacitive-controlled processes and diffusion-controlled processes, respectively. The value of k_1 can be obtained from the slopes of $v^{1/2}$ vs. $i/v^{1/2}$ plots.

Supplementary Figures and Tables

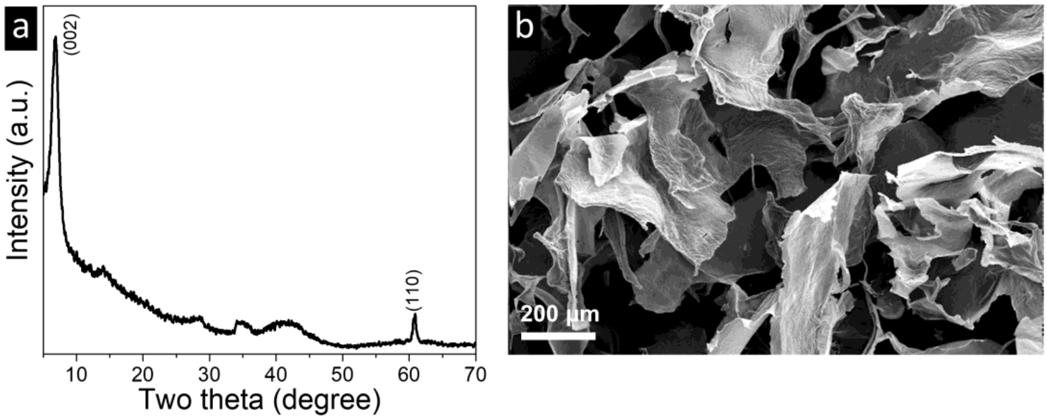

Figure S1: (a) XRD pattern and (b) SEM images of freeze-dried $Ti_3C_2T_z$ nanosheets. The $Ti_3C_2T_z$ nanosheets is collected by freeze-drying for 48 h at -61℃ via a lyophilizer (SP Virtis BenchTop Pro, US).

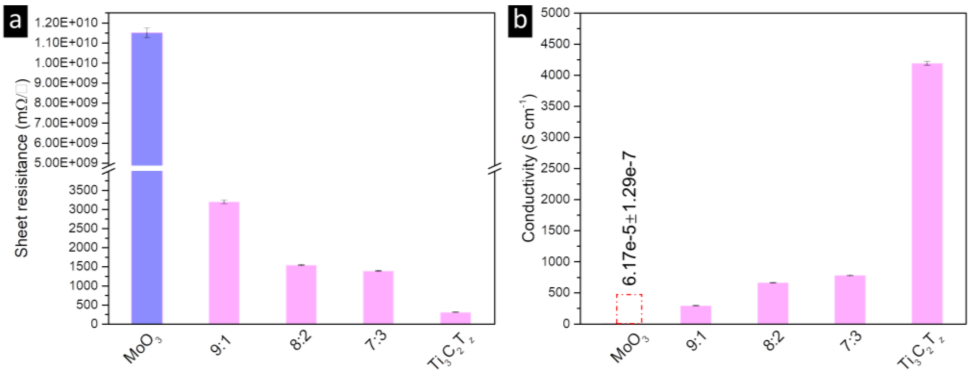

Figure S2: Sheet resistance (a) and conductivity (b) of MoO_3, $MoO_3/Ti_3C_2T_z$ and $Ti_3C_2T_z$ films, respectively.

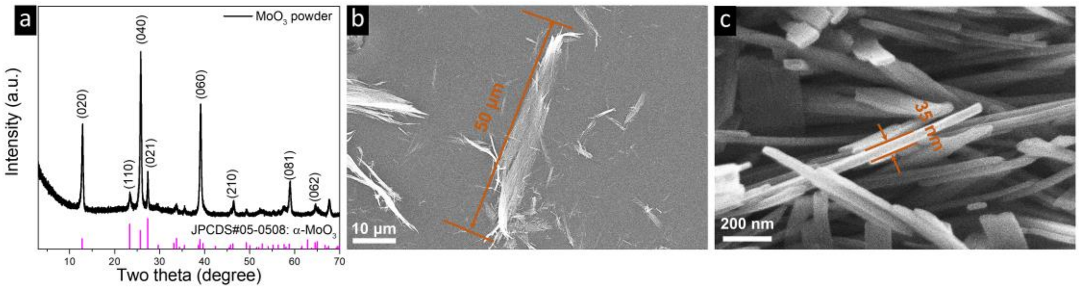

Figure S3: (a) XRD pattern, (b) and (c) SEM images of MoO_3 nanobelts. The lengths and thicknesses of the MoO_3 nanobelts (Figure S3b-c) are ≈ 50 μm and 35 nm, respectively, showing a high length-to-width ratio.

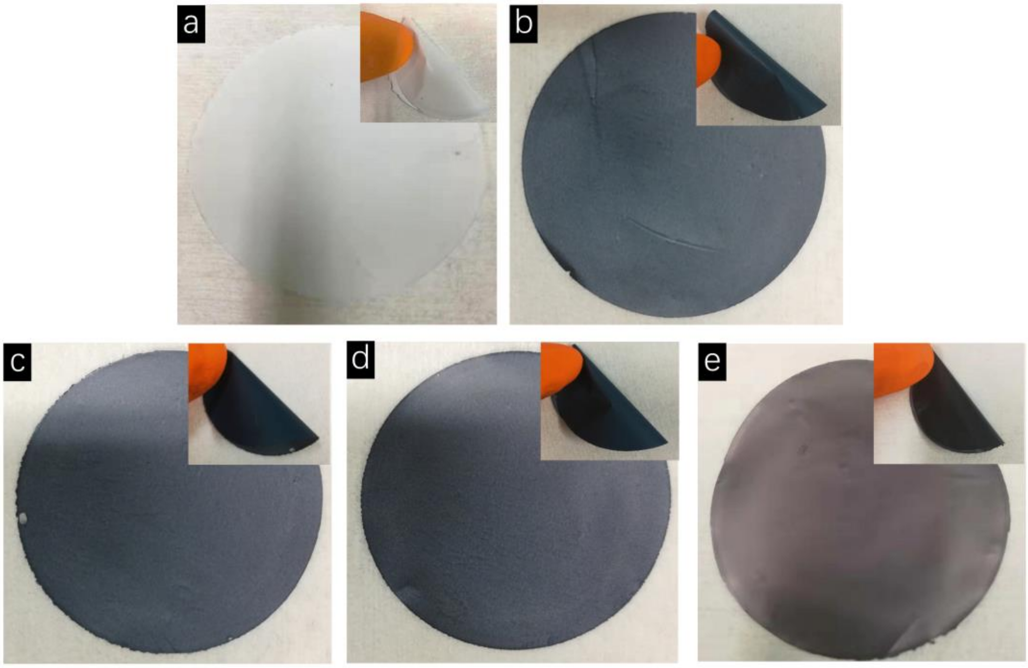

Figure S4. Digital photos of (a) pure MoO_3, (b) 9:1, (c) 8:2 (d) 7:3 and (e) pure $Ti_3C_2T_z$ films. Insets show their good flexibility.

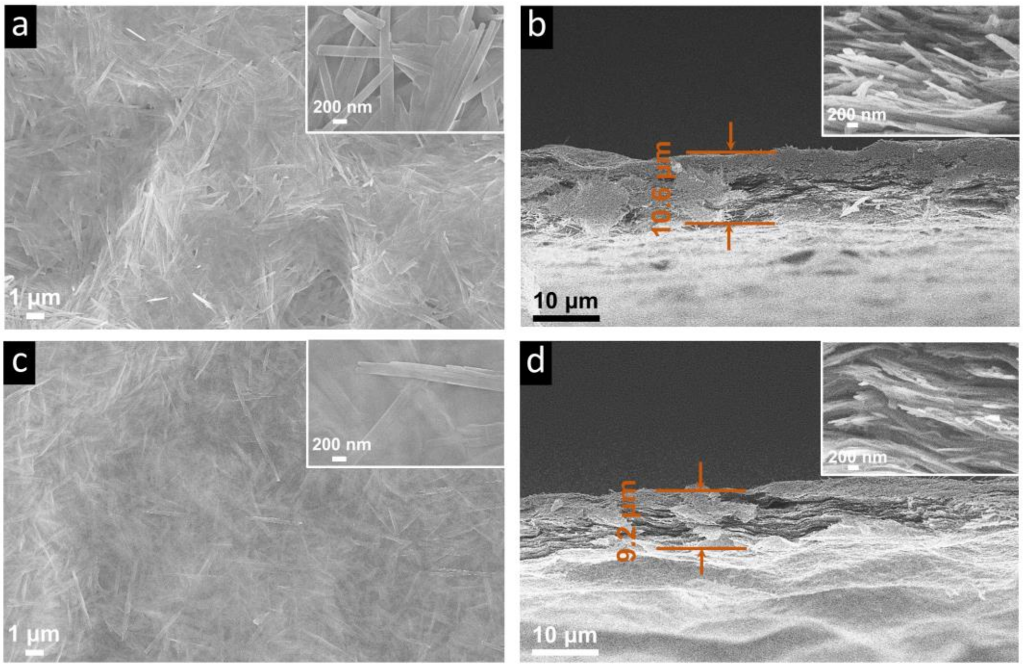

Figure S5. Top view and cross section of SEM images for (a, b) 9:1 and (c, d) 7:3 $MoO_3/Ti_3C_2T_z$ composite films

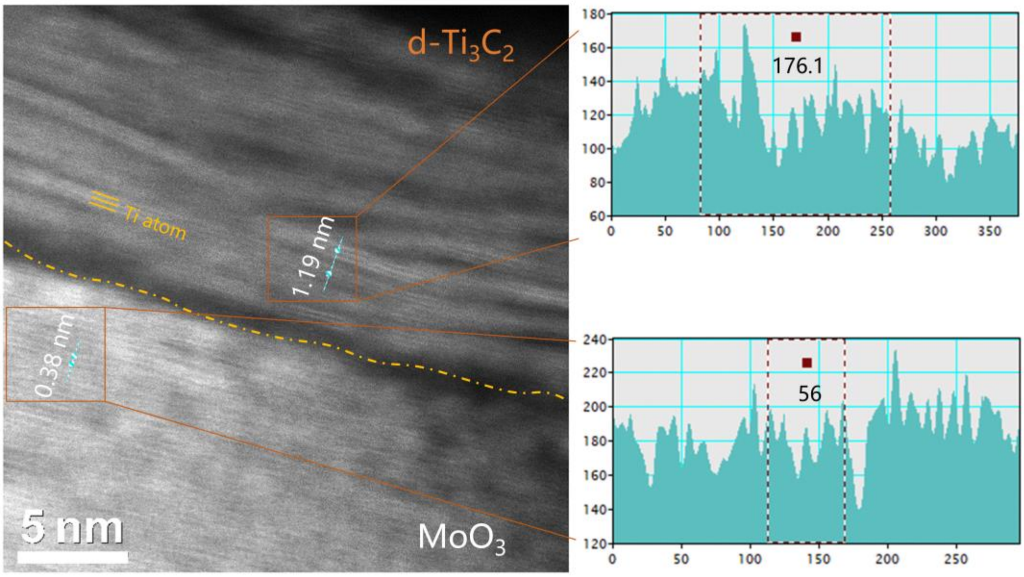

Figure S6: STEM cross-sectional image of 8:2 composite film is showing a lattice resolved image of the interface. The lattice fringes with interplanar distance of 1.19 nm and 0.38 nm correspond to the (002) and (001) crystal plane of $Ti_3C_2T_z$ and MoO_3, respectively.

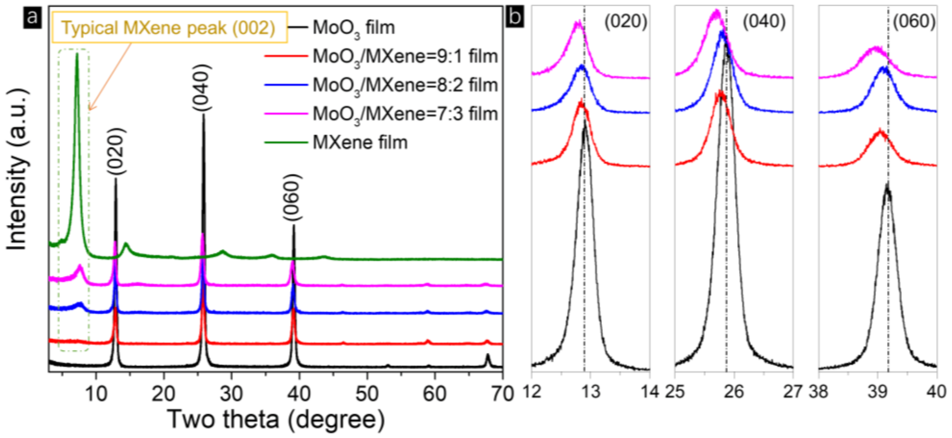

Figure S7: XRD patterns of MoO_3, $MoO_3/Ti_3C_2T_z$ and $Ti_3C_2T_z$ films. Based on the change in the position of (020) peak (in Figure S7b), the change in *b* lattice parameter of MoO_3 can be calculated, which increases from 13.73 Å for the MoO_3 film to 13.79 Å, 13.80 Å and 13.84 Å for the 9:1, 8:2 and 7:3 films, respectively. This means the increasing interlayer spacing of the MoO_3 results from the reduction, which may facilitate H^+ intercalation/de-intercalation.

Table S1: XPS peak fitting results of Mo 3d in MoO_3 and 8:2 $MoO_3/Ti_3C_2T_z$ films.

Sample	Region	Assigned to	BE [eV]	FWHM [eV]
MoO_3	Mo $3d_{5/2}(3d_{3/2})$	Mo^{6+}	232.7 (235.9)	0.85 (1.15)
8:2	Mo $3d_{5/2}(3d_{3/2})$	Mo^{6+}	232.7 (235.9)	1.07 (0.96)
		Mo^{5+}	231.7 (234.8)	1.03 (0.78)

Table S2: XPS peak fitting results of Ti 2p in $Ti_3C_2T_z$ and 8:2 $MoO_3/Ti_3C_2T_z$ films.

Sample	Region	Assigned to	BE [eV]	FWHM [eV]
$Ti_3C_2T_z$	Ti $2p_{3/2}$ ($2p_{1/2}$)	Ti-C	455.0 (460.8)	0.84 (1.25)
		Ti^{2+}	455.8 (461.4)	1.46 (1.49)
		Ti^{3+}	457.1 (462.7)	1.81 (2.29)
		TiO_2	459.0 (464.6)	1.60 (3.00)
8:2	Ti $2p_{3/2}$ ($2p_{1/2}$)	Ti-C	455.1 (461.2)	0.98 (1.40)
		Ti^{2+}	456.0 (460.6)	1.22 (2.41)
		Ti^{3+}	457.1 (462.3)	1.59 (1.73)
		TiO_2	459.0 (464.5)	1.31 (2.28)

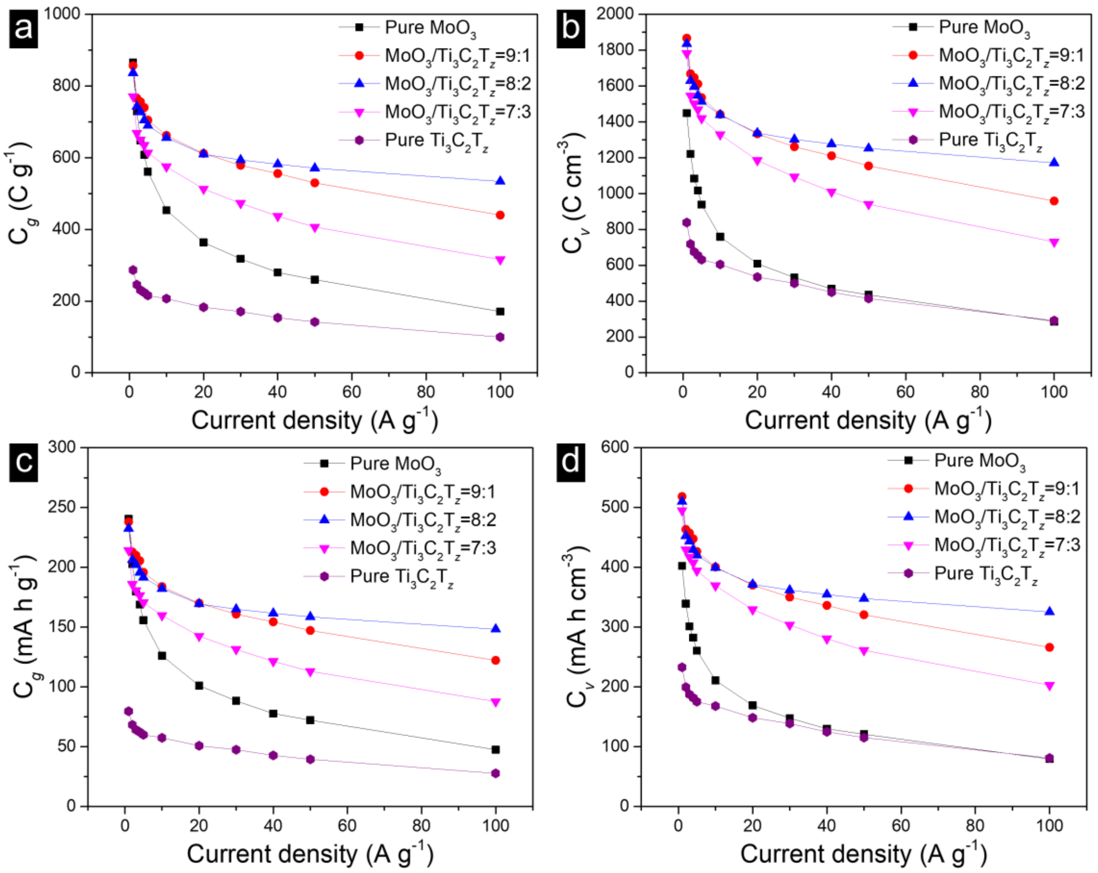

Figure S8: Specific capacities of MoO_3, $MoO_3/Ti_3C_2T_z$ composite and $Ti_3C_2T_z$ films at different current densities: C_g and C_v in (a) C g^{-1}, (b) C cm^{-3}, (c) mA h g^{-1} and (d) mA h cm^{-3}.

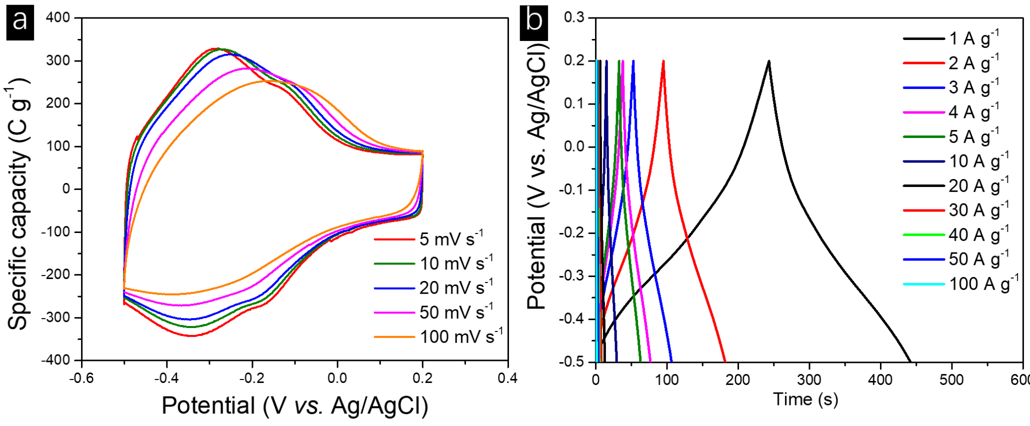

Figure S9: (a) CV curves, and (b) GCD curves of pure $Ti_3C_2T_z$ films.

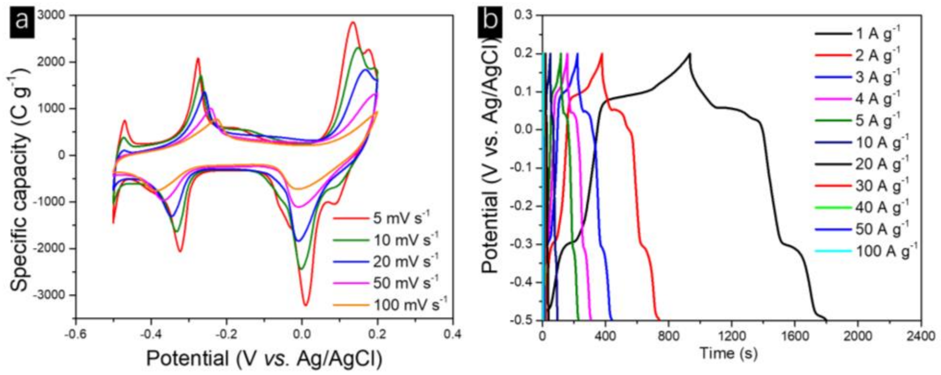

Figure S10: (a) CV and (b) GCD curves of pure MoO_3 films.

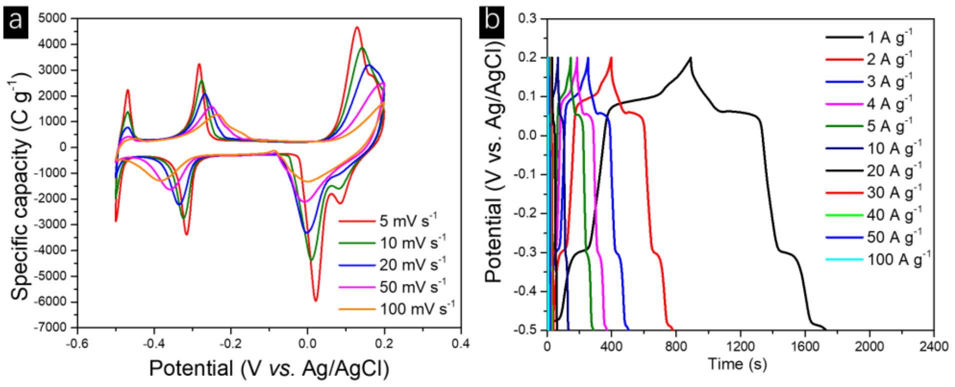

Figure S11: (a) CV and (b) GCD curves of 9:1 composite films.

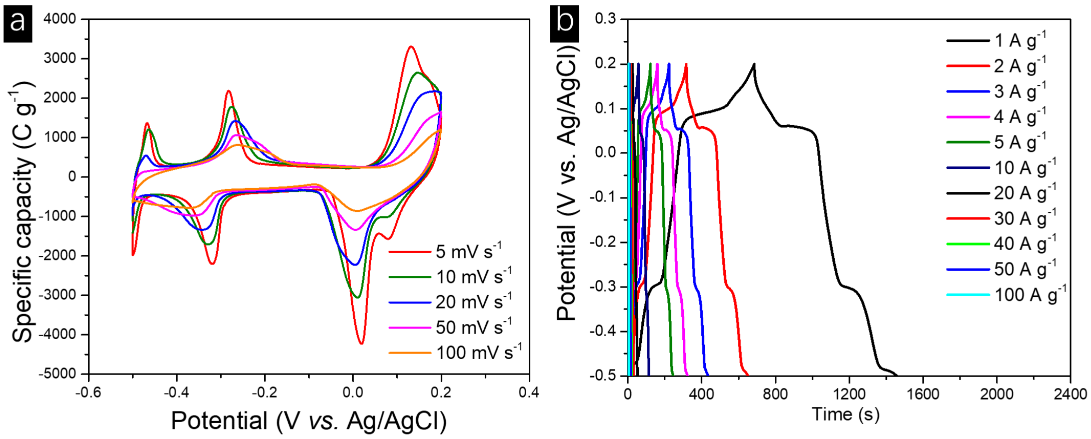

Figure S12: (a) CV, and (b) GCD curves of 7:3 films.

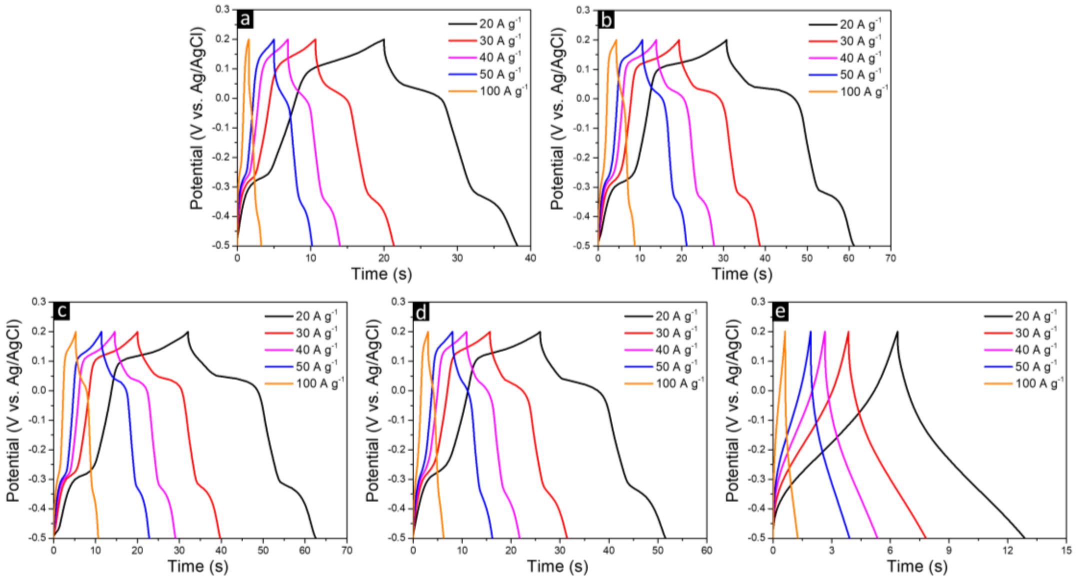

Figure S13: GCD curves of (a) MoO_3 (b) 9:1, (c) 8:2, (d) 7:3 and (e) $Ti_3C_2T_z$ films under high current densities.

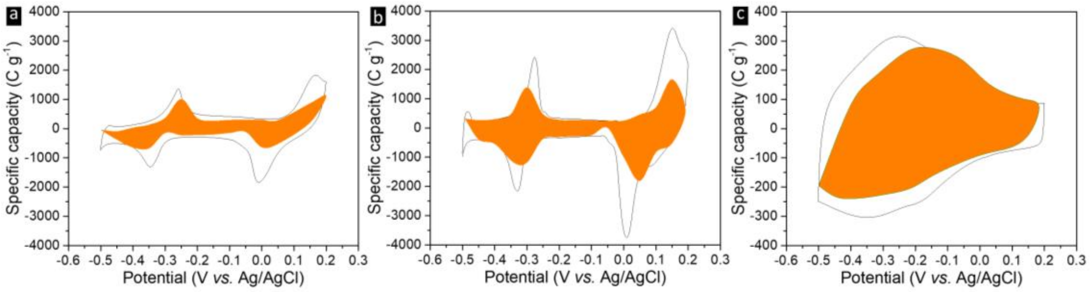

Figure S14: Comparison of capacitive-controlled contributions (shadow part) of the MoO_3, 8:2 and $Ti_3C_2T_z$ electrodes at 20 mV s^{-1}. The capacitive-controlled contributions are 55.1%, 68.3% and 80.8% for the MoO_3, 8:2 composite and $Ti_3C_2T_z$ electrodes, respectively.

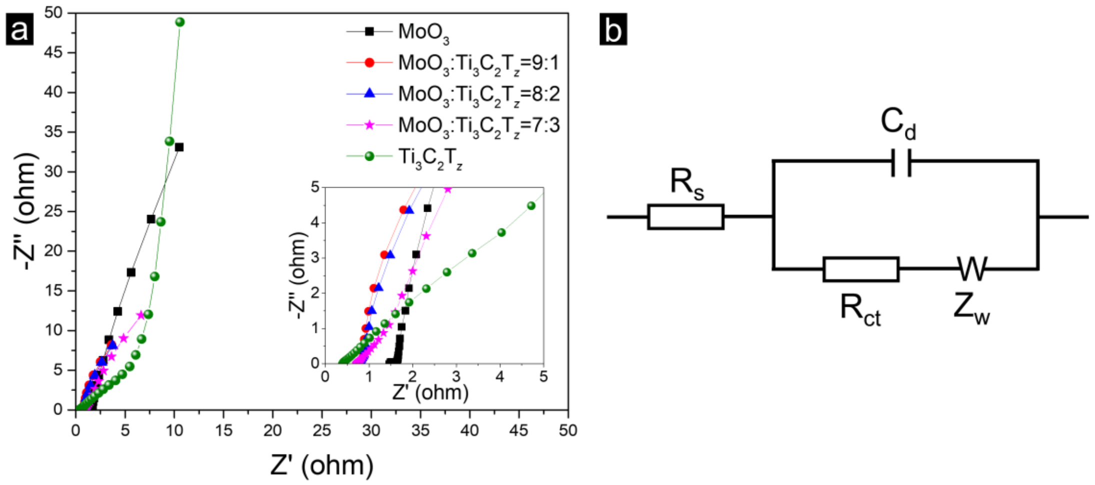

Figure S15: (a) Nyquist plots of MoO_3, $MoO_3/Ti_3C_2T_z$ composite and $Ti_3C_2T_z$ films. Inset is the magnified plot in the high frequency region of these electrodes. (b) The equivalent circuit of Nyquist plots for the five films. Electrochemical impedance spectroscopy (EIS) was conducted on a potentiostat (VSP, Biologic, France) in the frequency ranging from 0.01 to 100000 Hz with an alternating current amplitude of 5 mV. For the Nyquist plots, At high frequencies, the intersection point on the real axis reflects the internal resistance of the electrode (R_s);[2] the radius of semicircle on the Nyquist plot is related to the charge transfer resistance (R_{ct}).[3]

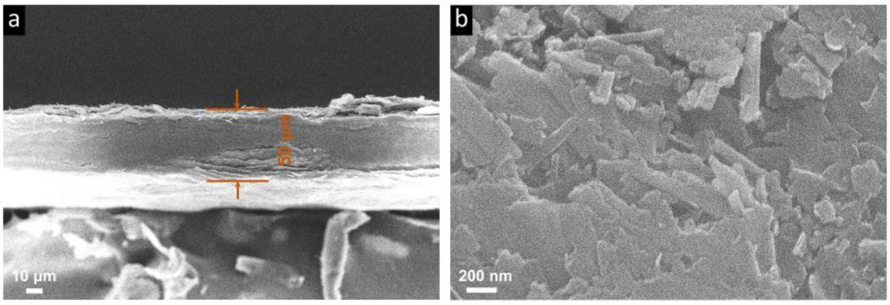

Figure S16: SEM images of 50 μm thick 8:2 composite film.

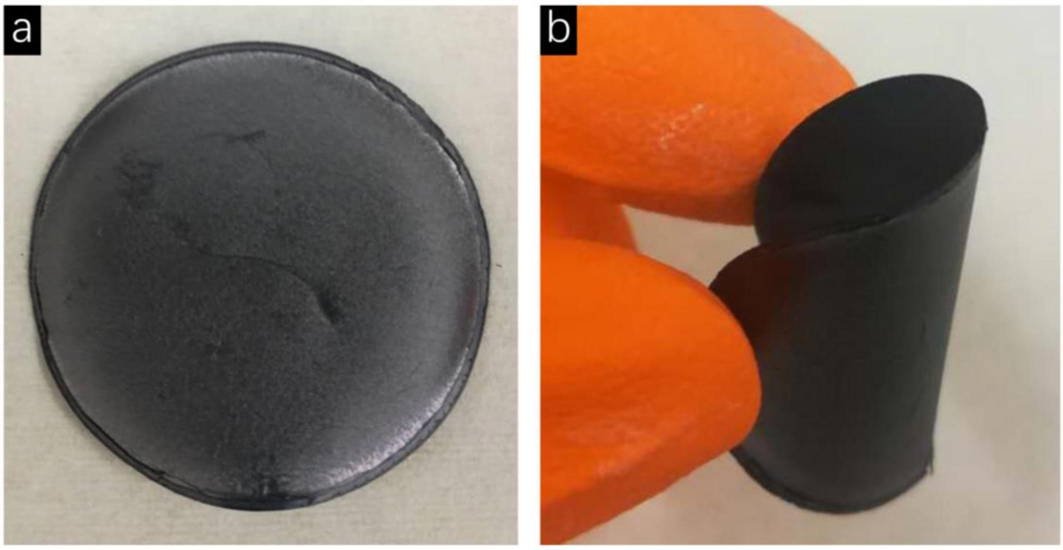

Figure S17: (a) Digital photo of image of 50 μm thick 8:2 composite film; (b) same as (a) but now bent near 180°.

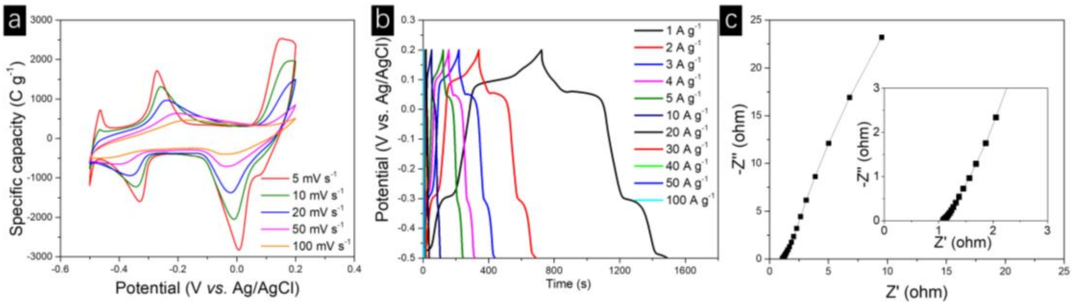

Figure S18: (a) CV, (b) GCD and (c) Nyquist plots of 50 μm thick 8:2 composite film.

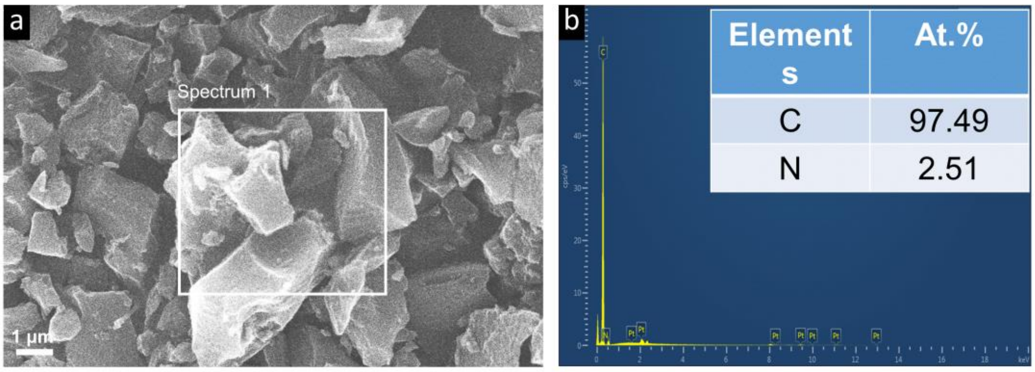

Figure S19: (a) SEM image and (b) EDX of NAC particles. Table inset in (b) shows 2.51 at.% of N in NAC particles, indicating that at least some N is doped into the AC.

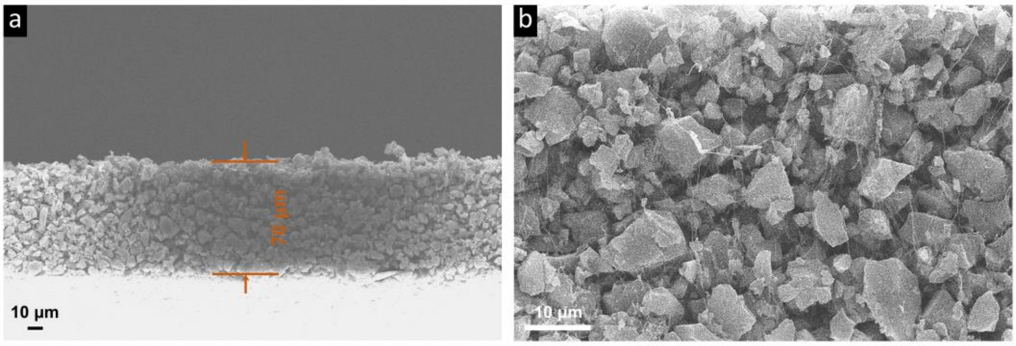

Figure S20: SEM images of NAC film with 10 wt.% PTFE.

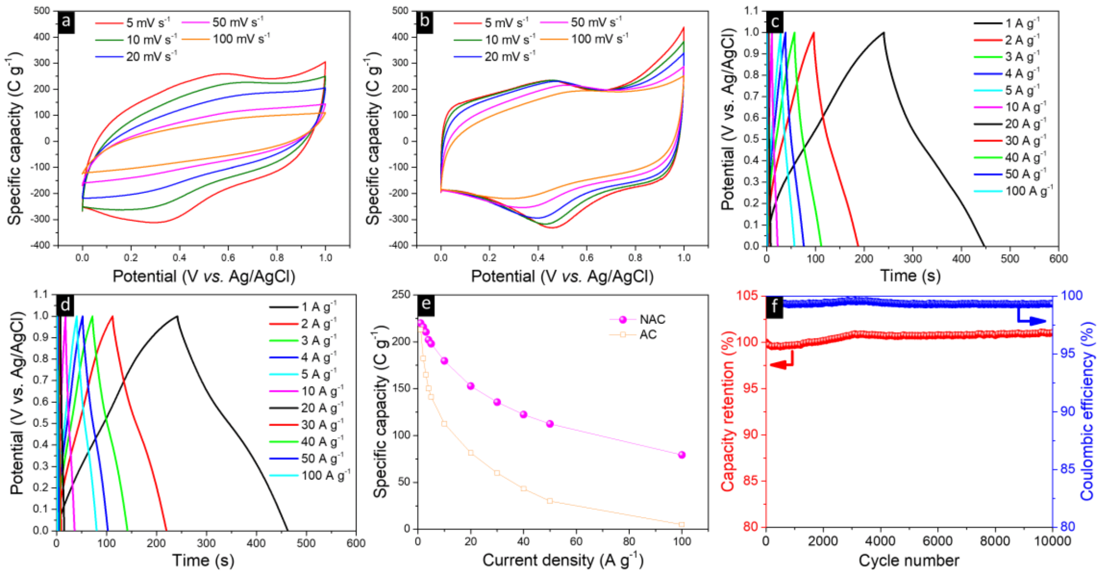

Figure S21: CV curves of (a) AC and (b) NAC; GCD curves of (c) AC and (d) NAC; (e) Specific capacities at different current densities of AC and NAC; (f) Cycling performance of NAC electrode at 20 A g^{-1} for 10,000 cycles. The rate performance of NAC is significantly enhanced after nitrogen-doping.

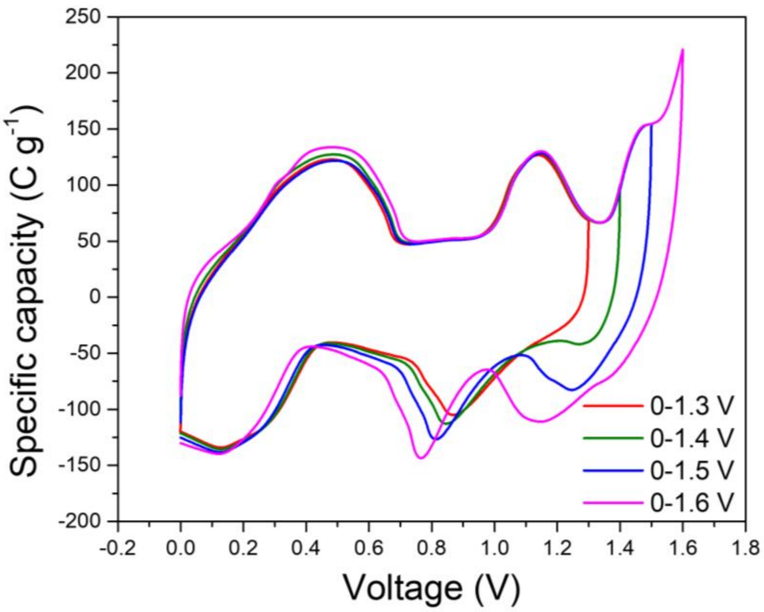

Figure S22: CV curves of 8:2//NAC hybrid capacitor at different potential windows.

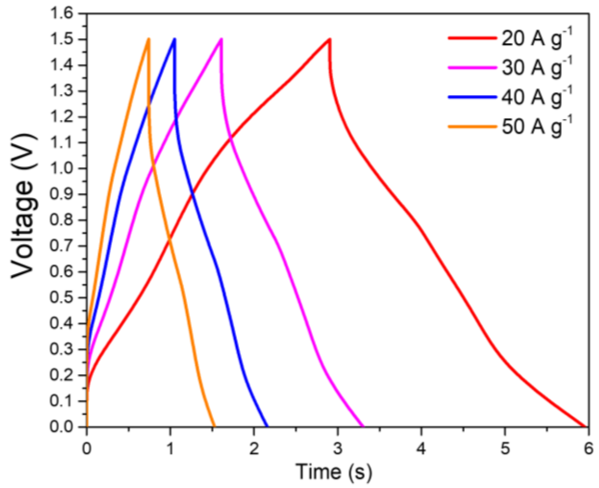

Figure S23: GCD curves at high current densities of 8:2//NAC hybrid capacitor.

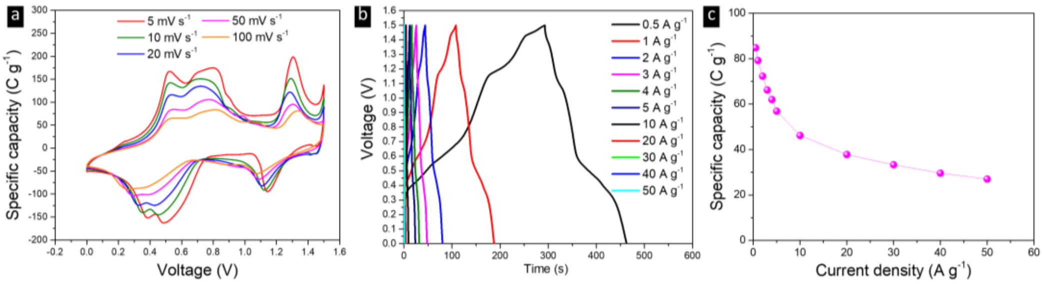

Figure S24: (a) CV, (b) GCD curves and (c) specific capacity at different current densities of MoO_3//NAC hybrid capacitor.

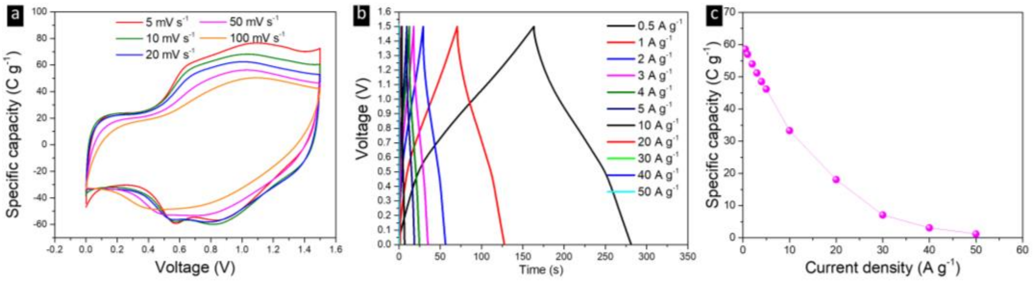

Figure S25: (a) CV, (b) GCD curves and (c) specific capacity at different current densities of $Ti_3C_2T_z$//NAC hybrid capacitor.

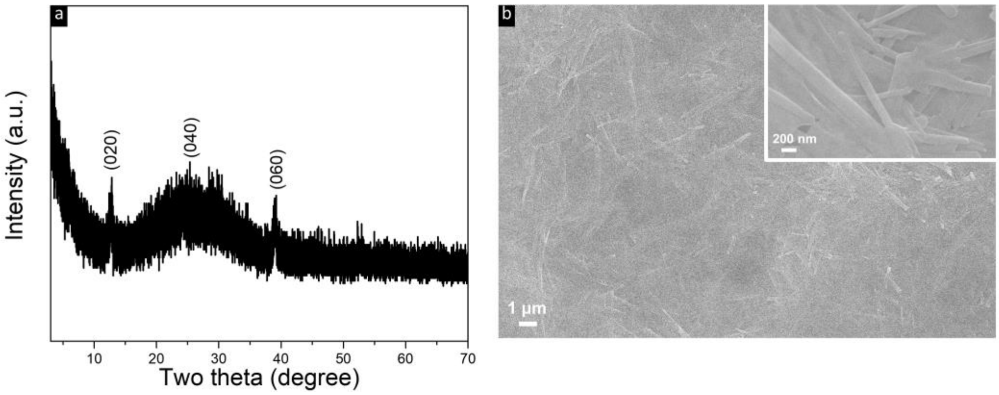

Figure S26: (a) XRD pattern and (b) SEM images of 8:2 composite film after 10,000 cycles in hybrid capacitor. Due to the small sized film, the background of peaks is strong. However, the (020), (040) and (060) peaks of MoO_3 can still be observed in Figure S26(a). Furthermore, the good film of $MoO_3/Ti_3C_2T_z$ is clear seen in the Figure S26b, indicating its robust structure.

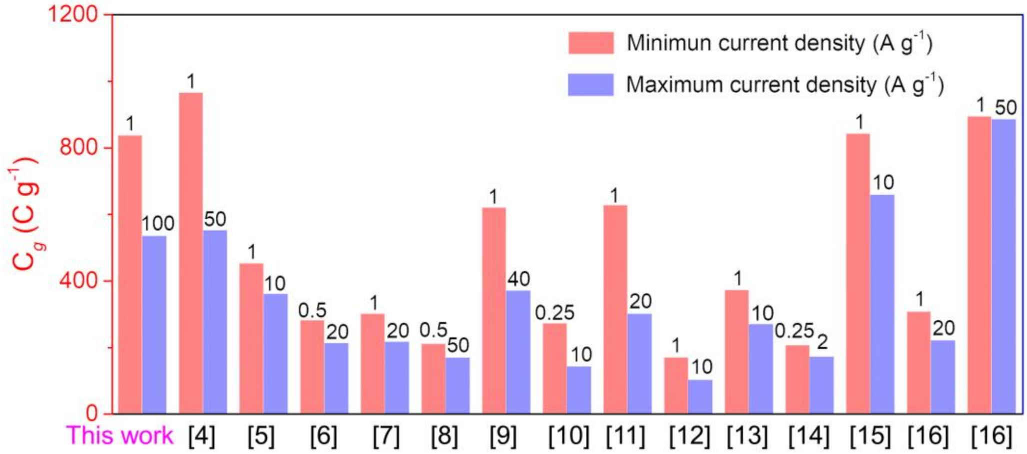

Figure S27: Comparison of the specific gravimetric capacities of the 8:2 composite electrodes (thin film, ≈ 10 μm) with previously reports, such as CoAl-LDH/MXene,[4] Ti_3C_2/polyaniline,[5] porous MXene film,[6] macroporous MXene film,[7] nitrogen-doped MXene,[8] polyaniline/rGO,[9] graphene/vanadium oxide film,[10] NiCoAl LDH/V_4C_3 MXene,[11] CuS/MXene,[12] $NiSe_2$/MXene,[13] MnO_2,[14] MOF derived LDH [15] and MoO_{3-x} nanobelts.[16]

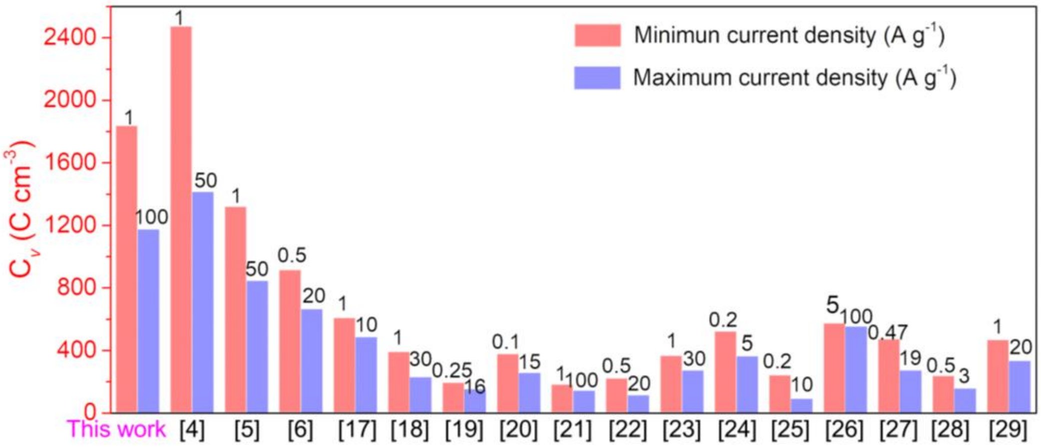

Figure S28: Comparison of specific volumetric capacities of the 8:2 composite electrodes (thin film, ≈ 10 μm), at two current densities, with previous reports, such as CoAl-LDH/MXene,[4] Ti_3C_2/polyaniline,[5] nanoporous MXene film,[6] MXene/PANI film,[17] organic molecules/graphene,[18] MnO_2/graphene,[19] porous carbon,[20] B, O-doped carbon nanofiber films,[21] N, P, S-doped porous carbon spheres,[22] polymer dots/graphene,[23] F, N-doped carbon microsphere,[24] PPy/GO,[25] graphene-polyaniline hydrogel,[26] polyaniline/graphene,[27] P, N-doped porous carbon [28] and holey graphene/PPy film.[29]

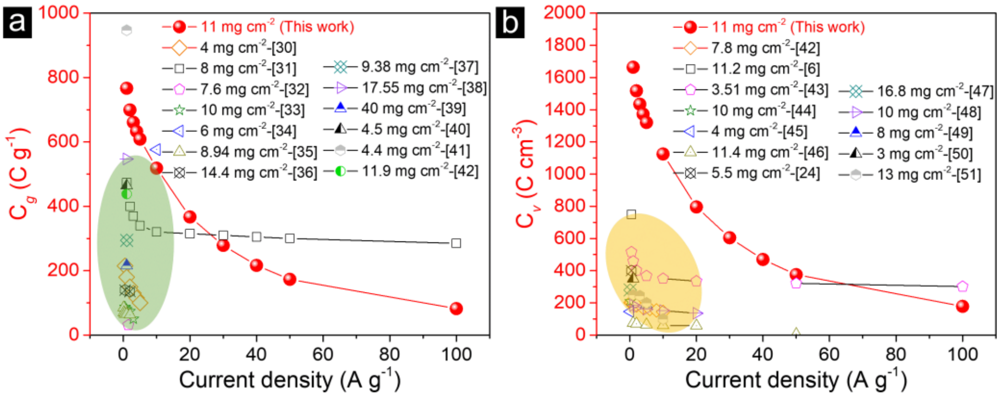

Figure S29: Comparison of the specific gravimetric capacity (a) and volumetric capacity (b) of the 8:2 (thickness ≈ 50 μm, mass loading ≈ 11 mg cm^{-2}) electrode with previously reports, such as graphene/carbon fiber,[30] doped carbon,[31] MXene film,[32] activated carbon fiber,[33] Co_3S_4 nanosheets,[34] graphene-PEDOT/PSS film,[35] porous carbon monolith,[36] NiO/carbon,[37] porous $Ni_3(NO_3)_2(OH)_4$ nanosheets,[38] doped carbon-tube,[39] MnO_2 nanograsses,[40] carbon-nickel cobalt sulphide [41] and $Ni(OH)_2$/graphene/bacterial cellulose;[42] $Ni(OH)_2$/graphene/bacterial cellulose,[42] porous MXene film,[6] vanadium nitride/carbon,[43] graphene,[44] carbon sphere,[45] graphene film,[46] F, N-doped carbon microsphere,[24] holey graphene,[47] graphene-carbon film,[48] nitrogen-doped porous carbon,[49] graphene-CNTs film,[50] RuO_2/graphene.[51]

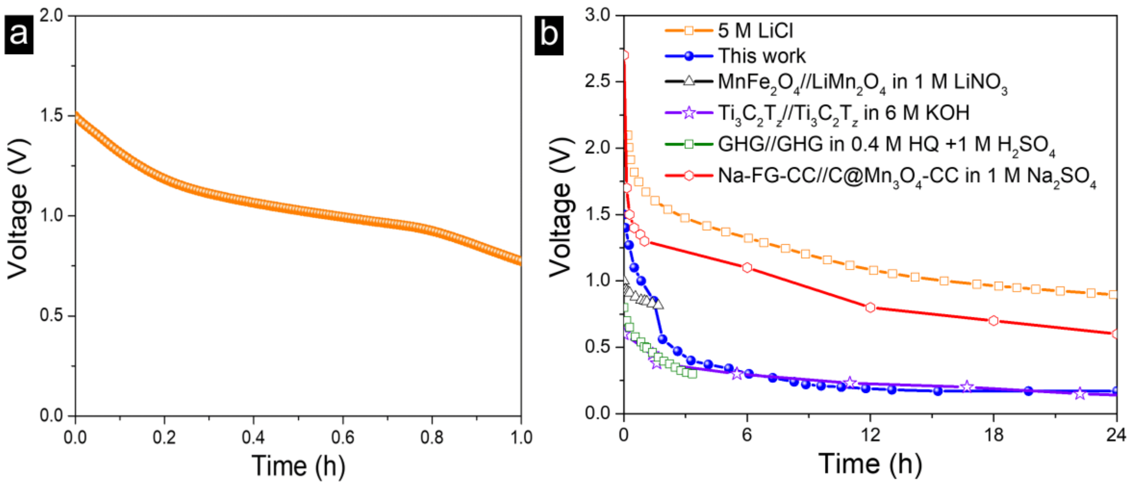

Figure S30: Self-discharge curves of 8:2//NAC device in (a) 1 h and (b) 24 h. The decay of the voltage was evaluated during 24 h, after consecutively charging the device to 1.5 V at 0.5 A g^{-1} and holding at 1.5 V for 2 h. Our results are compared with $MnFe_2O_4$//$LiMn_2O_4$ (Y.-P. Lin, N.-L. Wu, J. Power Sources **2011**, 196, 851), $Ti_3C_2T_z$//$Ti_3C_2T_z$ (Z. Wang, Z. Xu, H. Huang, X. Chu, Y. Xie, D. Xiong, C. Yan, H. Zhao, H. Zhang, W. Yang, ACS Nano **2020**, 14, 4916.), GHG//GHG (L. Chen, H. Bai, Z. Huang, L. Li, Energy Environ. Sci. **2014**, 7, 1750), and Na-FG-CC//C@Mn_3O_4-CC (X. Wu, B. Huang, Q. Wang, Y. Wang, J. Mater. Chem. A, **2019**, 7, 19017).

Self-discharge is not considered to be a significant issue for some consumer applications s (e.g. energy storage from regenerative breaking), while the electrochemical capacitor is stored in the charged state for long times (e.g. coupled with a battery in a cell phone), the impact on energy, power and recharging frequency of both the capacitor and battery can be significant.

The self-discharge rate in this work is compared with others shown in Figure S30. Clearly, our discharge rate would have to be greatly enhanced before any applications are contemplated. This comment notwithstanding, when we changed the electrolyte to 5 M LiCl in MoO_{3-x}/$Ti_3C_2T_z$//NAC cell, the self-discharge rate slowed down by almost as order of magnitude. We are currently trying to understand the origin of self-discharge in our systems in an attempt to slow it down. Tellingly, in the MXene-based supercapacitor literature, very few papers have reported the self-discharge rates of their devices.

Table S3: Comparison of mass loading and thicknesses of our results with those for different MXene-based films reported in the literature.

Samples	Mass loading (mg cm^{-2})	Thickness (μm)	Ref.
M-$Ti_3C_2T_z$ film	0.45	1.3	[52]
EG/$Ti_3C_2T_z$ film	<0.33	2.5	[53]
$Ti_3C_2T_z$ film	1.22	2.0	[54]
$Ti_3C_2T_z$/P-100-H film	<2.0	4.0	[55]
$Mo_{1.33}C$/PEDOT:PSS film	<2.0	2.0	[56]
UN-$Ti_3C_2T_z$ film	<1.0	3.0-4.0	[57]
N-$Ti_3C_2T_z$-300 film	1.2	6.1	[58]
3D $Ti_3C_2T_z$ film	<1	12	[59]
$Ti_3C_2T_z$ film	2-3	3	[60]
$Ti_3C_2T_z$/MPFs-6:1 film	<1	10-12	[61]
PPy@$Ti_3C_2T_z$ film	0.93	10	[62]
MoO_3/$Ti_3C_2T_z$ film	1	3	[63]
$Ti_3C_2T_z$/BC5 film	5	150	[64]
MoO_3/$Ti_3C_2T_z$ film	2.2	9.8	This work
MoO_3/$Ti_3C_2T_z$ film	11	51	This work

Table S4. Composition of films tested in this work.

Samples	MoO_3 nanobelts (weight percentage)	$Ti_3C_2T_z$ nanosheets (weight percentage)
MoO_3 film	100%	0
9:1 film	90%	10%
8:2 film	80%	20%
7:3 film	70%	30%
$Ti_3C_2T_z$ film	0	100%

References

[1] J. Li, X. Yuan, C. Lin, Y. Yang, L. Xu, X. Du, J. Xie, J. Lin, J. Sun, Adv. Energy Mater. 2017, 7, 1602725; R. Wang, S. Wang, Y. Zhang, D. Jin, X. Tao, L. Zhang, J. Mater. Chem. A 2017, 6, 1017; M. Sathiya, A. S. Prakash, K. Ramesha, J. M. Tarascon, A. K. Shukla, J. Am. Chem. Soc. 2011, 133, 16291.

[2] C. Yang, Y. Tang, Y. Tian, Y. Luo, Y. He, X. Yin, W. Que, Adv. Funct. Mater. 2018, 1705487.

[3] C. Zhao, W. Qian, Z. Huang, S. Passerini, X. Qian, ACS Appl. Mater. Interfaces 2016, 8, 15661.

[4] H. Niu, X. Yang, Q. Wang, X. Jing, K. Cheng, K. Zhu, K. Ye, G. Wang, D. Cao, J. Yan, J. Energy Chem. 2020, 46, 105.

[5] S. Wang, Z. Ma, Q.-F. Lü, H. Yang, ChemElectroChem 2019, 6, 2748.

[6] Z. Fan, Y. Wang, Z. Xie, X. Xu, Y. Yuan, Z. Cheng, Y. Liu, Nanoscale 2018, 10, 9642.

[7] M. Yao, Y. Chen, Z. Wang, C. Shao, J. Dong, Q. Zhang, L. Zhang, X. Zhao, Chem. Eng. J. 2020, 124057.

[8] C. Yang, Y. Tang, Y. Tian, Y. Luo, X. Yin, W. Que, ACS Appl. Energy Mater. 2020, 3, 586.

[9] K. Jin, W. Zhang, Y. Wang, X. Guo, Z. Chen, L. Li, Y. Zhang, Z. Wang, J. Chen, L. Sun, T. Zhang, Electrochim. Acta 2018, 285, 221.

[10] L. Deng, Y. Gao, Z. Ma, G. Fan, J. Colloid. Interf. Sci. 2017, 505, 556.

[11] X. Wang, H. Li, H. Li, S. Lin, J. Bai, J. Dai, C. Liang, X. Zhu, Y. Sun, S. Dou, J. Mater. Chem. A 2019, 7, 2291.

[12] Z. Pan, F. Cao, X. Hu, X. Ji, J. Mater. Chem. A 2019, 7, 8984.

[13] H. Jiang, Z. Wang, Q. Yang, L. Tan, L. Dong, M. Dong, Nano-Micro Lett. 2019, 11, 31.

[14] Y. Guo, L. Li, L. Song, M. Wu, Y. Gao, J. Chen, C. Mao, J. Song, H. Niu, J. Mater. Chem. A 2019, 7, 12661.

[15] Z. Jiang, Z. Li, Z. Qin, H. Sun, X. Jiao, D. Chen, Nanoscale 2013, 5, 11770.

[16] J. Yang, X. Xiao, P. Chen, K. Zhu, K. Cheng, K. Ye, G. Wang, D. Cao, J. Yan, Nano Energy 2019, 58, 455; Q.-L. Wu, S.-X. Zhao, L. Yu, X.-X. Zheng, Y.-F. Wang, L.-Q. Yu, C.-W. Nan, G. Cao, J. Mater. Chem. A 2019, 7, 13205.

[17] A. VahidMohammadi, J. Moncada, H. Chen, E. Kayali, J. Orangi, C. A. Carrero, M. Beidaghi, J. Mater. Chem. A 2018, 6, 22123.
[18] L. Zhang, D. Han, Y. Tao, C. Cui, Y. Deng, X. Dong, W. Lv, Z. Lin, S. Wu, Z. Weng, Q.-H. Yang, J. Mater. Chem. A 2020, 8, 461.
[19] X. Zhao, L. Zhang, S. Murali, M. D. Stoller, Q. Zhang, Y. Zhu, R. S. Ruoff, ACS Nano 2012, 6, 5404.
[20] Y. Tao, X. Xie, W. Lv, D.-M. Tang, D. Kong, Z. Huang, H. Nishihara, T. Ishii, B. Li, D. Golberg, F. Kang, T. Kyotani, Q.-H. Yang, Sci. Rep. 2013, 3, 2975.
[21] Z.-Y. Yu, L.-F. Chen, L.-T. Song, Y.-W. Zhu, H.-X. Ji, S.-H. Yu, Nano Energy 2015, 15, 235.
[22] L. Yan, D. Li, T. Yan, G. Chen, L. Shi, Z. An, D. Zhang, ACS Sustainable Chem. Eng. 2018, 6, 5265.
[23] J.-S. Wei, J. Chen, H. Ding, P. Zhang, Y.-G. Wang, H.-M. Xiong, J. Power Sources 2017, 364, 465.
[24] J. Zhou, J. Lian, L. Hou, J. Zhang, H. Gou, M. Xia, Y. Zhao, T. A. Strobel, L. Tao, F. Gao, Nat. Commun. 2015, 6, 8503.
[25] Z. Wang, P. Tammela, M. Strømme, L. Nyholm, Nanoscale 2015, 7, 3418.
[26] Y. Wang, X. Yang, A. G. Pandolfo, J. Ding, D. Li, Adv. Energy Mater. 2016, 6, 1600185.
[27] J. Pedrós, A. Boscá, J. Martínez, S. Ruiz-Gómez, L. Pérez, V. Barranco, F. Calle, J. Power Sources 2016, 317, 35.
[28] X. Yan, Y. Yu, S.-K. Ryu, J. Lan, X. Jia, X. Yang, Electrochim. Acta 2014, 136, 466.
[29] Z. Fan, J. Zhu, X. Sun, Z. Cheng, Y. Liu, Y. Wang, ACS Appl. Mater. Interfaces 2017, 9, 21763.
[30] Y. Huang, C. Shen, Z. Tang, T. Shi, S. Zheng, L. Lin, ChemElectroChem 2019, 6, 6009.
[31] N. Mao, H. Wang, Y. Sui, Y. Cui, J. Pokrzywinski, J. Shi, W. Liu, S. Chen, X. Wang, D. Mitlin, Nano Res. 2017, 10, 1767.
[32] S.-Y. Lin, X. Zhang, J. Power Sources 2015, 294, 354.
[33] M. Vijayakumar, R. Santhosh, J. Adduru, T. N. Rao, M. Karthik, Carbon 2018, 140, 465.
[34] B. Xin, Y. Zhao, C. Xu, J. Solid State Electr. 2016, 20, 2197.
[35] Y. Liu, B. Weng, J. M. Razal, Q. Xu, C. Zhao, Y. Hou, S. Seyedin, R. Jalili, G. G. Wallace,

J. Chen, Sci. Rep. 2015, 5, 17045.
[36] H. Li, D. Yuan, C. Tang, S. Wang, J. Sun, Z. Li, T. Tang, F. Wang, H. Gong, C. He, Carbon 2016, 100, 151.
[37] K. Tao, P. Li, L. Kang, X. Li, Q. Zhou, L. Dong, W. Liang, J. Power Sources 2015, 293, 23.
[38] M. Shi, M. Cui, L. Kang, T. Li, S. Yun, J. Du, S. Xu, Y. Liu, Appl. Surf. Sci. 2018, 427, 678.
[39] J. Zhao, Y. Li, G. Wang, T. Wei, Z. Liu, K. Cheng, K. Ye, K. Zhu, D. Cao, Z. Fan, J. Mater. Chem. A 2017, 5, 23085.
[40] H. Wang, C. Xu, Y. Chen, Y. Wang, Energy Stor. Mater. 2017, 8, 127.
[41] H. Wang, C. Wang, C. Qing, D. Sun, B. Wang, G. Qu, M. Sun, Y. Tang, Electrochim. Acta 2015, 174, 1104.
[42] L. Ma, R. Liu, L. Liu, F. Wang, H. Niu, Y. Huang, J. Power Sources 2016, 335, 76.
[43] Q. Li, Y. Chen, J. Zhang, W. Tian, L. Wang, Z. Ren, X. Ren, X. Li, B. Gao, X. Peng, P. K. Chu, K. Huo, Nano Energy 2018, 51, 128.
[44] X. Yang, C. Cheng, Y. Wang, L. Qiu, D. Li, Science 2013, 341, 534.
[45] S. Feng, Z. Liu, Q. Yu, Z. Zhuang, Q. Chen, S. Fu, L. Zhou, L. Mai, ACS Appl. Mater. Interfaces 2019, 11, 4011.
[46] G. Lian, C.-C. Tuan, L. Li, S. Jiao, K.-S. Moon, Q. Wang, D. Cui, C.-P. Wong, Nano Lett. 2017, 17, 1365.
[47] Y. Xu, Z. Lin, X. Zhong, X. Huang, N. O. Weiss, Y. Huang, X. Duan, Nat. Commun. 2014, 5, 4554.
[48] N. Díez, M. Qiao, J. L. Gómez-Urbano, C. Botas, D. Carriazo, M. M. Titirici, J. Mater. Chem. A 2019, 7, 6126.
[49] T. Ouyang, K. Cheng, Y. Gao, S. Kong, K. Ye, G. Wang, D. Cao, J. Mater. Chem. A 2016, 4, 9832.
[50] N. Díez, C. Botas, R. Mysyk, E. Goikolea, T. Rojo, D. Carriazo, J. Mater. Chem. A 2018, 6, 3667.
[51] H. Ma, D. Kong, Y. Xu, X. Xie, Y. Tao, Z. Xiao, W. Lv, H. D. Jang, J. Huang, Q.-H. Yang, Small 2017, 13, 1701026.

[52]K. Li, X. Wang, S. Li, P. Urbankowski, J. Li, Y. Xu, Y. Gogotsi, Small 2020, 16, 1906851.
[53]H. Li, Y. Hou, F. Wang, M. R. Lohe, X. Zhuang, L. Niu, X. Feng, Adv. Energy Mater. 2017, 7, 1601847.
[54]A. M. Navarro-Suárez, K. L. Van Aken, T. Mathis, T. Makaryan, J. Yan, J. Carretero-González, T. Rojo, Y. Gogotsi, Electrochim. Acta 2018, 259, 752.
[55]L. Li, N. Zhang, M. Zhang, X. Zhang, Z. Zhang, Dalton Trans. 2019, 48, 1747.
[56]L. Qin, Q. Tao, A. El Ghazaly, J. Fernandez-Rodriguez, P. O. Å. Persson, J. Rosen, F. Zhang, Adv. Funct. Mater. 2017, 1703808.
[57]C. Yang, Y. Tang, Y. Tian, Y. Luo, M. F. U. Din, X. Yin, W. Que, Adv. Energy Mater. 2018, 8.
[58]Y. Tian, W. Que, Y. Luo, C. Yang, X. Yin, L. B. Kong, J. Mater. Chem. A 2019, 7, 5416.
[59]X. Zhang, X. Liu, S. Dong, J. Yang, Y. Liu, Appl. Mater. Today 2019, 16, 315.
[60]M. R. Lukatskaya, O. Mashtalir, C. E. Ren, Y. Dall'Agnese, P. Rozier, P. L. Taberna, M. Naguib, P. Simon, M. W. Barsoum, Y. Gogotsi, Science 2013, 341, 1502.
[61]W. Zhao, J. Peng, W. Wang, B. Jin, T. Chen, S. Liu, Q. Zhao, W. Huang, Small 2019, 15.
[62]M. Boota, B. Anasori, C. Voigt, M.-Q. Zhao, M. W. Barsoum, Y. Gogotsi, Adv. Mater. 2016, 28, 1517.
[63]Y. Wang, X. Wang, X. Li, R. Liu, Y. Bai, H. Xiao, Y. Liu, G. H. Yuan, Nano-Micro Letters 2020, 12, 115.
[64]Y. Wang, X. Wang, X. Li, Y. Bai, H. Xiao, Y. Liu, R. Liu, G. Yuan, Adv. Funct. Mater. 2019, 29, 1900326.
